# *Parvimonas micra* activates the Ras/ERK/c-Fos pathway by upregulating miR-218-5p to promote colorectal cancer progression

**DOI:** 10.1186/s13046-022-02572-2

**Published:** 2023-01-10

**Authors:** Yuxiao Chang, Ziran Huang, Fengyi Hou, Yuejiao Liu, Likun Wang, Zhen Wang, Yifan Sun, Zhiyuan Pan, Yafang Tan, Lei Ding, Hong Gao, Ruifu Yang, Yujing Bi

**Affiliations:** 1 State Key Laboratory of Pathogen and Biosecurity, Beijing Institute of Microbiology and Epidemiology, Beijing, 100071 China; 2 Department of Colorectal Surgery, Department of General Surgery, Beijing Shijitan Hospital, Capital Medical University, Beijing, 100038 China

**Keywords:** Parvimonas micra, CRC, Exosomes, microRNA, Colorectal cancer, Ras/ERK/c-Fos signaling pathway

## Abstract

**Background:**

Colorectal cancer (CRC) is the third most common cancer in the world, and a strong relationship exists between CRC and gut microbiota, which affects the occurrence, development, and metastasis of cancer. Bioinformatics-based analyses revealed that the abundance of *Parvimonas micra* (*P. micra*) in the feces of patients with cancer is significantly higher than that in healthy people. Therefore, an important relationship may exist between *P. micra* and CRC.

**Methods:**

We first confirmed that *P. micra* can promote the proliferation of cell lines through cell experiments and mouse models. Then we selected the signaling pathways and content of exosomes to promote the development of CRC by transcriptomics and microRNA sequencing. Finally, we confirmed that *P. micra* promoted CRC development through miR-218-5p/Ras/ERK/c-Fos pathway through the in vivo and in vitro experiments.

**Results:**

First, it was confirmed by in vitro and in vivo experiments that *P. micra* can promote the development of CRC. Transcriptome analysis after the coincubation of bacteria and cells revealed that *P. micra* promoted cell proliferation by activating the Ras/ERK/c-Fos pathway. Furthermore, microRNA sequencing analysis of the cells and exosomes showed that miR-218-5p and protein tyrosine phosphatase receptor R (PTPRR) were the key factors involved in activating the Ras/ERK/c-Fos pathway, and the miR-218-5p inhibitor was used to confirm the role of microRNA in xenograft mice.

**Conclusion:**

This experiment confirmed that *P. micra* promoted the development of CRC by upregulating miR-218-5p expression in cells and exosomes, inhibiting *PTPRR* expression, and ultimately activating the Ras/ERK/c-Fos signaling pathway.

**Supplementary Information:**

The online version contains supplementary material available at 10.1186/s13046-022-02572-2.

## Introduction

Colorectal cancer (CRC) is the third most common cancer in the world and tends to affect younger individuals. The morbidity of CRC in people under 50 years of age is rising rapidly [1, 2]. Many factors affect the occurrence of CRC, such as external environment, diet, genetic factors, internal immune, and gut microbiota [3, 4]. However, the pathogenesis of CRC is unclear. The gut microbiota is closely related to CRC. In addition to *Fusobacterium nucleatum*, *Enterotoxigenic Bacteroides fragilis*, and *PKS*^*+*^ *Escherichia coli*, which have been confirmed earlier [5–7], in recent years, various bacteria, such as *Porphyromonas gingivalis*, *Peptostreptococcus anaerobius*, and *Clostridium difficile* have been confirmed to be associated with CRC [8–10].

*P. micra* is an oral opportunistic pathogen, which occurs frequently in oral diseases. *P. micra* can form biofilms by itself, and can also assist other pathogens in forming biofilms, leading to inflammation or disease [11, 12]. In recent years, it has been found that *P. micra* is not only an oral opportunistic pathogen, but also may be closely related to CRC. Many bioinformatics-based studies have reported that *P. micra* is closely involved in CRC [13–15], and can be used as a marker for CRC diagnosis [16, 17]. However, the relationship between *P. micra* and CRC has not been confirmed experimentally.

MicroRNA is a family of RNAs that can negatively regulate the expression of genes. The main function of microRNAs is to regulate the expression of genes related to growth, development, and disease occurrence and development. MicroRNAs play an important role in cardiovascular disease, diabetes, Alzheimer’s disease, Parkinson’s disease, and many other diseases, especially tumors. In CRC, specific microRNAs may play important roles in bacteria-promoting cancer development [18, 19]. Functional differences between different tumor types and cancer stages were associated with microRNAs expression. Alterations in microRNA expression are associated with the expression of genes essential for cell proliferation or survival, leading to tumor formation. For example, overexpression of miR-494 induced Wnt/β-catenin signaling by targeting APC, thus promoting CRC cell growth [20]. Moreover, some can be used as biomarkers for the diagnosis, prognosis, and prediction of metastasis in patients with CRC [21]. In this study, we confirmed that *P. micra* can promote the development of CRC in vivo and in vitro. Furthermore, *P. micra* can regulate the production and secretion of miR-218-5p, and then promote the development of CRC through the Ras/ERK/c-Fos pathway. These findings provide diagnostic and therapeutic targets for treating CRC.

## Methods

### Sample

#### Bacterial strains

Culture samples (colorectal cancer mucosal tissues) were taken from the Beijing Shijitan Hospital. Isolation and culture were performed as described [22]. A strain of *Parvimonas micra* was isolated from the sample and named *Pm-*42. DH5α strain were purchase from Sangon Biotech Co., Ltd. (Shanghai, China).

#### Colorectal cancer mucosa and adjacent normal tissue

The 22 patients with CRC included in this study were from Shijitan Hospital, Beijing. We took 2 cm^2^ colorectal cancer mucosal tissue and adjacent normal tissue samples, at least 5 cm away from the cancerous tissue.

#### Cell lines

LoVo and HT-29 cells were purchased from Beina Biological Co., Ltd. (Henan, China) .

### Cell counting

LoVo or HT-29 cells were seeded into a 24-well plate. A total of 1 × 10^4^ cells per well were incubated at 37 °C for 4 h in 5% CO_2_. Then, *Pm-*42 or DH5α strains were added to the plate at a multiplicity of infection (MOI) of 10. The plates were incubated in an anaerobic incubator for 2 h and then at 37 °C for 24 and 48 h in 5% CO_2_. The cells seeded in the wells were counted with a cell counter (Invitrogen, Countess 3 FL). PBS was used as a blank control.

### MTT cell proliferation assay

LoVo or HT-29 cells were seeded in a 96-well plate. A total of 2 × 10^4^ cells per well were incubated at 37 °C for 4 h in 5% CO_2_; *Pm-*42 or DH5α cells were added to the plate at an MOI of 10, that is, 2 × 10^5^ per well. To evaluate proliferation, the MTT cell proliferation and cytotoxicity assay kit (Solarbio, M1020) was used 24 and 48 h after the coincubation, and absorbance was detected at 490 nm. PBS was used as a blank control.

### Real-time cell analysis (RTCA)

A total of 2 × 10^4^ LoVo or HT-29 cells were seeded into the wells of a plate. After 8 h, *Pm-*42 or DH5α was added to the well at an MOI of 10. The plate was placed on the machine for 48 h. PBS was used as a blank control.

### Animal experiments

#### Xenograft model

We purchased 6-week-old female nude mice from Vital River Laboratory Animal Technology (Beijing, China). LoVo or HT-29 cells were incubated with *Pm-*42 or DH5α strains at an MOI of 10 for 24 h. The cells were collected and washed three times with PBS, mixed with *Pm-*42 or DH5α at an MOI of 1:1, and injected subcutaneously into nude mice. The number of cells administered to each mouse was 3 × 10^6^ cells/mouse. Three hours after the injection, the mice were given an intraperitoneal injection of piperacillin antibiotic therapy (150 mg/kg body weight). PBS was used as a blank control. Body weight and tumor volume were subsequently measured every two days. On the 24th day after modeling, the mice were killed and the blood was taken, and then the serum was extracted for cytokine detection. Cytokines were detected with the Bio-Plex 200® system (Bio-Rad, United States).

Tumor-bearing nude mice treated with the antagomir (GenePharma, China) were subcutaneously injected with cells using the same protocol. After nine days of cell injection, 5 nmol antagomir was injected locally into the tumor every three days for a total of five injections. Body weight and tumor volume were measured every two days throughout the experiment.

#### *P. micra* gavage administration in APC^Min/+^ mice

APC^Min/+^ mice were 5-week-old females purchased from GemPharmatech Biotechnology Co., Ltd. (Nanjing, China). Mice were fed streptomycin (2 mg/mL) in water for three days before gavage. Mice were given 10^9^ CFU bacteria once a day by gavage for 12 weeks. Blood and feces were collected at the midpoint or endpoint of gavage. Serum biochemistry was performed using a biochemical detector (Beckman Coulter, AU480), and cytokines were detected with the Bio-Plex 200® system (Bio-Rad, United States).

### Pathological and immunohistochemical detection

Fresh tissues were fixed in 4% paraformaldehyde for over 24 h. Tissues were embedded in paraffin. For pathological detection, the paraffin-embedded colonic sections (5 μm thick) were stained with hematoxylin and eosin. For immunohistochemical (IHC) detection, the sections were stained with anti-ERK1/2 (Cell Signaling Technology, 4695 T), anti-c-Fos (Abcam, ab222699), and anti-PTPRR (Abcam, ab180134) antibodies.

### Cells transcriptome sequencing

LoVo cells were incubated with *Pm-*42 or PBS for 48 h at an MOI of 10. After incubation, the cells were collected, and washed three times with PBS. Cellular RNA was extracted with the PureLink RNA Mini Kit (Invitrogen, 12183018a). The RNA samples were sent to Novogene Biological Technology Company (Beijing, China) for transcriptome sequencing. Raw data were uploaded to GenBank (BioProject PRJNA859257).

### Cells microRNA sequencing

HT-29 cells were incubated with *Pm-*42 or PBS for 48 h at an MOI of 10. After incubation, the cells were collected and washed three times with PBS. Cellular RNA was extracted by using Trizol (Ambion, 15,596,026). Finally, the RNA samples were sent to Novogene Biological Technology Company (Beijing, China) for microRNA sequencing. Raw data were uploaded to GenBank (BioProject PRJNA859413 and PRJNA859414).

### Cell exosomes sequencing

HT-29 cells were cultured for 24 h. The medium was replaced with serum-free media. The cells were incubated with *Pm-*42 or PBS at an MOI of 10 for 48 h. Cell supernatants were collected, and exosomes were extracted using the Extracellular vesicle extraction kit (Raojing Gene, China), followed by RNA extraction with the miRNeasy Mini Kit (QIAGEN, 217004). Library construction was performed with the NEBNext Multiplex Small RNA Sample Prep Set for Illumina (NE, E7300). Raw data were uploaded to GenBank (BioProject PRJNA859256).

### DNA extraction and 16S rDNA sequencing of mice feces

Fresh feces were collected from mice at the end of gavage. DNA was extracted from feces by using the QIAamp PowerFecal Pro DNA kit (QIAGEN, 51804). The extracted DNA was sent to Novogene Biological Technology Company (Beijing, China) for 16S rDNA sequencing. Raw data were uploaded to GenBank (BioProject PRJNA859250).

### Western blot

Total protein of cells or tissues was separated using sodium dodecyl sulfate polyacrylamide gel electrophoresis (SDS-PAGE). The proteins in SDS-PAGE were transferred onto a nitrocellulose membrane (Millipore). The membrane was incubated first with primary antibodies and then with secondary antibodies. The primary and secondary antibodies used are as follows: anti-Ras (Abcam, ab108602), anti-ERK1/2 (Cell Signaling Technology, 4695 T), anti-p-ERK1/2 (Cell Signaling Technology, 4370 T), anti-c-Fos (Abcam, ab222699), anti-PTPRR (Abcam, ab180134), anti-β-actin (Ray antibody, EM2001), and a fluorescent secondary antibody (LICOR, 926–32,211 and 926–32,210). The band intensity ratio of the target protein to β-actin was calculated. Fold change = (target protein/β-actin)/mean value of the band intensity ratio in the blank group. The experiment was performed in three independent replicates.

### Quantitative polymerase chain reaction (qPCR)

Cell samples were directly collected and washed with PBS three times. Mucosal tissue samples were homogenized (HODER, China). RNA was extracted with the Trizol reagent. Mucosal tissues were homogenized and treated with lysozyme (Thermo Scientific, 89,833) for four hours. Total DNA was extracted with the QIAamp DNA mini Kit (QIAGEN, 51306). Total RNA, containing mRNAs and microRNAs, was converted to cDNA using reverse transcription reagents (Vazyme, R323–01 and MR101–01) according to the manufacturer’s protocols. qPCR-based detection of *P. micra,* mRNA and microRNA was performed using SYBR Green qPCR Master Mix (Vazyme, Q511–02) in the LightCycler 480 II Real-Time PCR System (Roch, Switzerland). Data were presented as fold change using the 2^-△△CT^ method. Primer sequences are listed in Supplemental Table 1.

### Transfection of plasmid, mimics, inhibitor, and negative control into the cells

Cells were seeded into wells and transfected with the pcDNA3.1(+)-hPTPRR or pcDNA3.1(+) using a transfection reagent (Vazyme, T101–01), or transfected with the mimics, inhibitor, or negative control (NC) using a transfection reagent (Polyplus, 101,000,028). Bacteria were added for coincubation using the method used in MTT cell proliferation assay, 24 h after transfection.

### Prediction of the miR-218-5p target genes and the binding sites to the target genes

Target genes were screened using a target gene prediction database. The starBase database integrates the prediction results of seven databases to predict microRNA target genes (https://starbase.sysu.edu.cn/). Target genes predicted in ≥ 4 databases are screened out. The binding sites for miR-218-5p and protein tyrosine phosphatase receptor R (PTPRR) were predicted using TargetScan (https://www.targetscan.org/).

### Luciferase reporter assay

To construct the dual luciferase plasmid, we cloned: 1) the 3′-untranslated region (3′-UTR) of the target site (of the target gene corresponding to the microRNA) and its adjacent sequences into the pmirGlo vector as the wild type and 2) mutated 3′-UTR target site (of the target gene corresponding to the microRNA) into the pmirGlo vector as the mutant type. HT-29 or LoVo cells were cultured in 12-well plates until the cell density reached ~ 60%. The cells were cotransfected with the pmirGlo plasmid and mimics or mimics negative control using a transfection reagent (Vazyme, T101–01), and the luciferase assay was performed using the Dual Luciferase Reporter Assay Kit (Vazyme, DL101–01) according to the manufacturer’s protocols. Relative luciferase activity = (Firefly/Renilla) / mean (Firefly/Renilla) of the mimics NC.

### The Cancer genome atlas database analysis

The Cancer Genome Atlas (TCGA) is a project overseen by the National Cancer Institute and the National Human Genome Research Institute to apply high-throughput genome analysis techniques to help understand cancer (https://portal.gdc.cancer.gov/). We used the Xiantao academic (https://www.xiantao.love/) to search the TCGA database for transcriptome sequencing and microRNA sequencing data related to colorectal cancer and to analyze the data and prepare figures. Screened TCGA database ID numbers can be found in Supplemental Table 2.

### Statistical analysis

For comparisons between multiple sets of data, the normality and homogeneity of variance of each data set were first confirmed. If the data were distributed normally and showed homogeneity of variance, analysis of variance (ANOVA) or two-tailed unpaired Student’s t-test was used, whereas if they were not normally distributed or showed heterogeneity of variance, a rank-sum test was used (*P* < 0.05). For the comparison of *P. micra* abundance and genes (or microRNA) expression in tumor and adjacent normal tissues, a paired *T* test was used for statistical analysis. *P. micra* abundance and gene (or microRNA) expression were statistically calculated using Spearmans’s correlation analysis. Data are expressed as mean ± SD from 3 independent experiments. All *P* values were 2-tailed and P values of < 0.05 were considered statistically significant (*P < 0.05; **P < 0.01; ***P < 0.001). All statistical analyses were done using the SPSS Statistics 20.0 software.

## Results

### *P. micra* promotes CRC proliferation in the cell lines and xenograft animal model

**Fig. 1:**
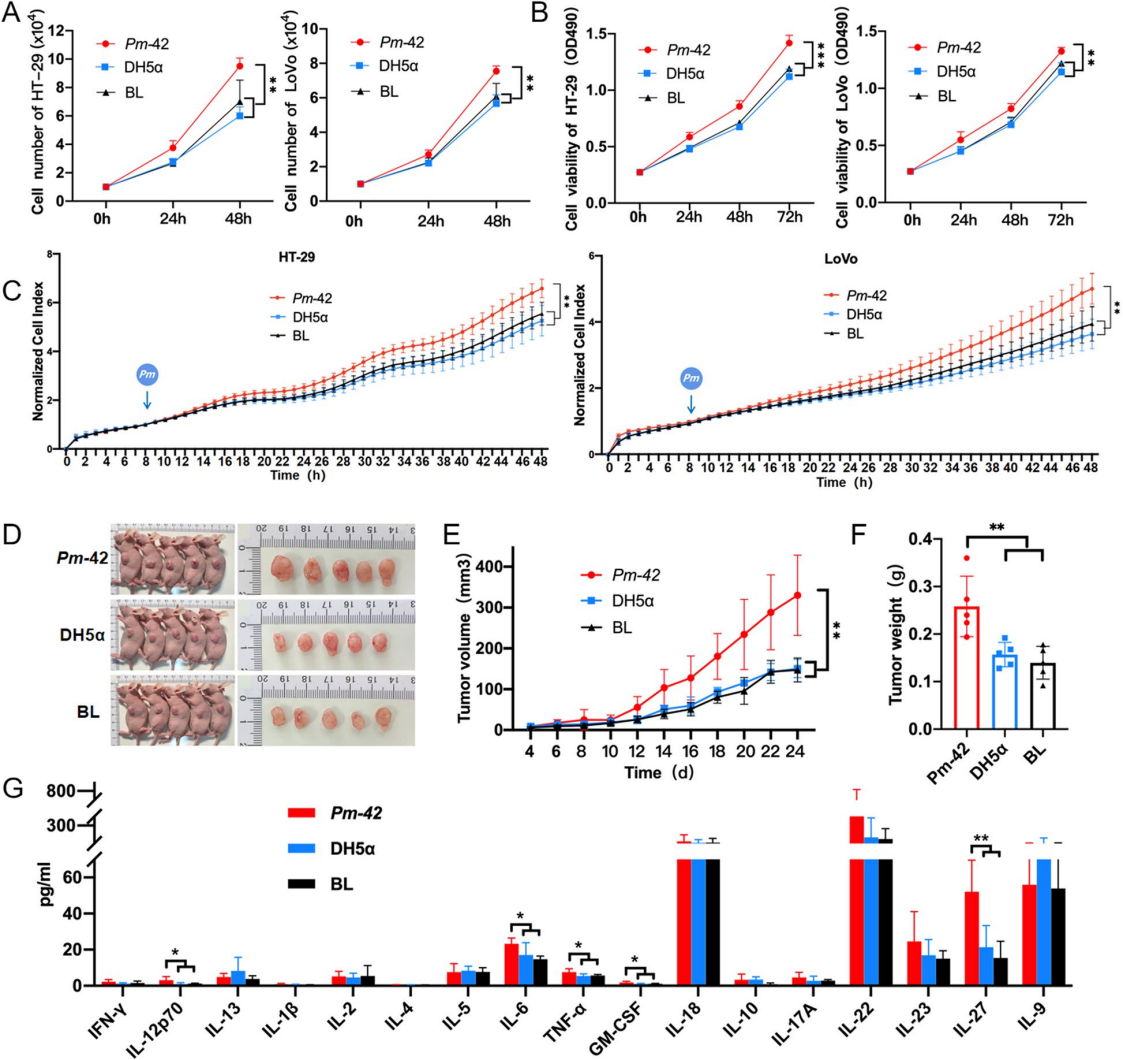
*Parvimonas micra* promotes cancer cell proliferation. **A**–**C** Detection of *P. micra* promoting cancer cell line proliferation through cell counting, MTT assay, and real-time cell analysis. Data are expressed as mean ± SD from 3 independent experiments. **D**–**G** LoVo cells treated with *Pm-*42, DH5α, or PBS were subcutaneously injected into male BALB/C nude mice to produce xenograft tumors in animals. Data are expressed as mean ± SD, *n* = 5. **D** Images of xenograft mice. **E** The change of tumor size in xenograft mice. **F** The weight of tumor in xenograft mice. **G** The detection of 17 cytokines in the sera of xenograft mice

We selected two CRC epithelial cell lines, LoVo and HT-29. DH5α has been used as a negative control in many in vitro experiments demonstrating the relationship between bacteria and colorectal cancer [18, 23]. Therefore, cells were infected with *Pm-42* and DH-5α for 24 and 48 h. *Pm-*42 significantly stimulated cell proliferation compared with other groups (Fig. 1A). Results from the MTT assay were similar (Fig. 1B). RTCA was used to evaluate the effects of *Pm-*42 on epithelial cells, and the results were consistent with the cell count and MTT assays (Fig. 1C). Next, we used a xenograft model in nude mice to confirm that *P. micra* can promote the proliferation of CRC cells in vivo. The results showed that the tumor volume of the *Pm-*42 group was significantly higher than that of the other two groups. No significant difference was observed between the DH5α and blank groups (Fig. 1D, Figure1E and Supplemental Fig. 1A). There was also a significant difference in tumor weight between the *Pm-*42 group and the other two groups (Fig. 1F); however, no significant difference was present in the spleen index (Supplemental Fig. 1B). Furthermore, evaluation of 17 types of cytokines in mice sera revealed that levels of IL-6, TNF-α, IL-12p70, GM-CSF, and IL-27 in the *Pm-*42 group were significantly higher than those in the other two groups (Fig. 1G). These results suggest that *P. micra* can promote the proliferation of CRC cells.

### *P. micra* promotes tumor proliferation and alters the gut microbiota in APC^Min/+^ mice

**Fig. 2:**
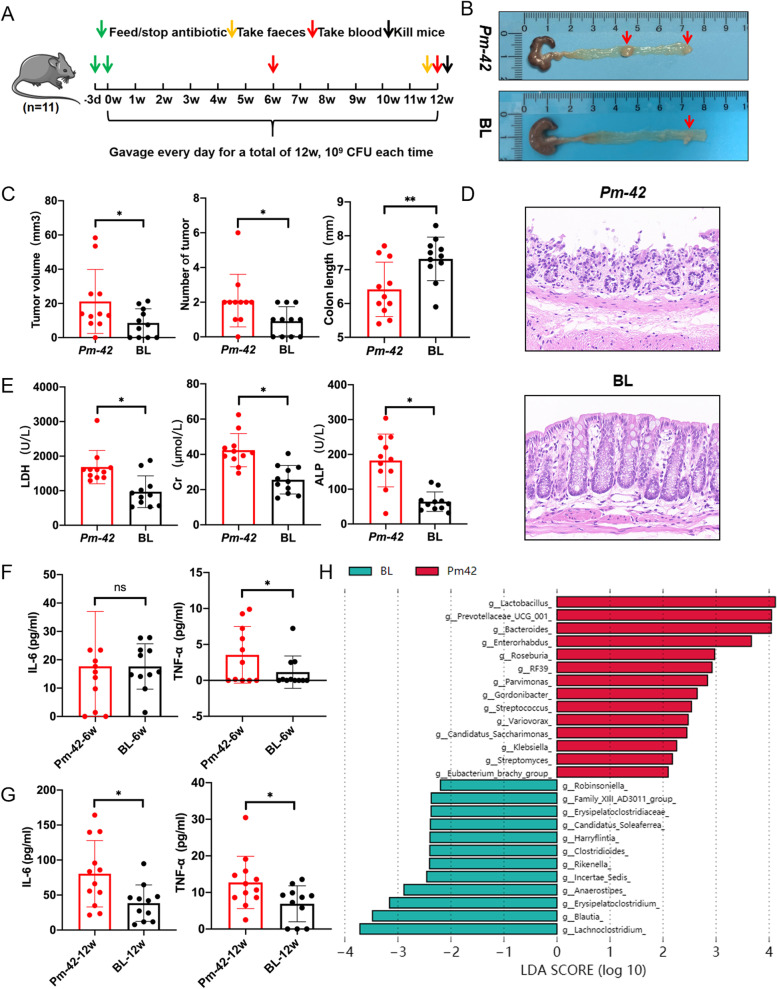
*Parvimonas micra* promotes colorectal cancer in APC^Min/+^ mice. **A** APC^Min/+^ mice were administrated with *P. micra* or PBS for 12 weeks, and fecal collection was performed at the end of gavage. Blood was collected at the midpoint and endpoint of gavage. **B** Representative images of APC^Min/+^ mice colon. **C** Changes in tumor size, tumor number, and colon length in APC^Min/+^ mice. **D** Representative images of colon pathology. **E** Serum biochemical assay of APC^Min/+^ mice. **F** Serum cytokine detection in mice at midpoint (6 weeks). **G** Serum cytokine detection in mice at endpoint (12 weeks). **H** Linear discriminant analysis effect size analysis of the gut microbiota with 16S rDNA sequencing. Data are expressed as mean ± SD, *n* = 11

APC^Min/+^ mice are commonly used as intestinal tumor models. We used APC^Min/+^ mice to perform the *P. micra* gavage experiment. The mice were first given antibiotic–water (streptomycin) for three days, and then *Pm-*42 at 1 × 10^9^ CFU or PBS by gavage daily for the next 12 weeks. Feces were collected at the end of gavage, and blood was collected at the midpoint and endpoint (Fig. 2A). Both anatomical results (Fig. 2B) and results from the analysis of tumor volume, tumor number, and colon length (Fig. 2C) revealed that the *Pm-*42 group had significantly more serious phenotypes than the blank group. Pathological results showed that the epithelial cells of the colon mucosa in the *Pm-*42 group were atrophic, necrotic, and exfoliated; the glands were incomplete; and inflammatory cells infiltration was more extensive (Fig. 2D). Blood biochemistry results revealed that in the *Pm-*42 group, levels of lactate dehydrogenase (LDH), creatinine (Cr), and alkaline phosphatase (ALP) were significantly higher than those in the blank group (Fig. 2E). Cytokine analysis revealed that in the *Pm-*42 group, TNF-α was significantly higher than that in the blank group after 6-weeks of gavage (Fig. 2F), in the *Pm-*42 group, IL-6 and TNF-α were significantly higher than those in the blank group at the end of 12-weeks of gavage (Fig. 2G). The level of cytokines at 12-weeks was higher than that at 6-weeks, indicating that inflammation was continuously increasing.

To observe changes in the gut microbiota, at the end of gavage we collected feces from APC^Min/+^ mice and performed sequencing. In terms of abundance composition at the genus level, a significant difference between the *Pm-*42 and blank groups was observed (Supplemental Fig. 2A and B), and as seen in the Wayne diagram, the unique species between the two groups accounted for 50.9% and 31.8% of the total species in each group, respectively (Supplemental Fig. 2C). In terms of β-diversity, results from principal coordinate analysis (PCoA) revealed a significant difference between the two groups (Supplemental Fig. 2D). In addition, linear discriminant analysis effect size (LEfSe) analysis identified 14 differential bacteria, including *Parvimonas*, at the genus level in the *Pm-*42 group (Fig. 2H). Of these, *Prevotellaceae*, *Streptococcus*, *Klebsiella*, and *Streptomyces* are positively associated with gastrointestinal inflammation or cancer [24–27]. The results of the Metastat analysis were consistent with LEfSe (Supplemental Fig. 3A and B). In addition, we verified the abundance of *P. micra* in the two groups by qPCR, which showed no difference between the two groups before the experiment and a significant difference between the two groups after the experiment (Supplemental Fig. 3C and D). Taken together, these results suggest that *P. micra* can colonize the gut and change the composition of the gut microbiota.

### *P. micra* promotes tumor proliferation through the Ras/ERK/c-Fos signaling pathway

**Fig. 3:**
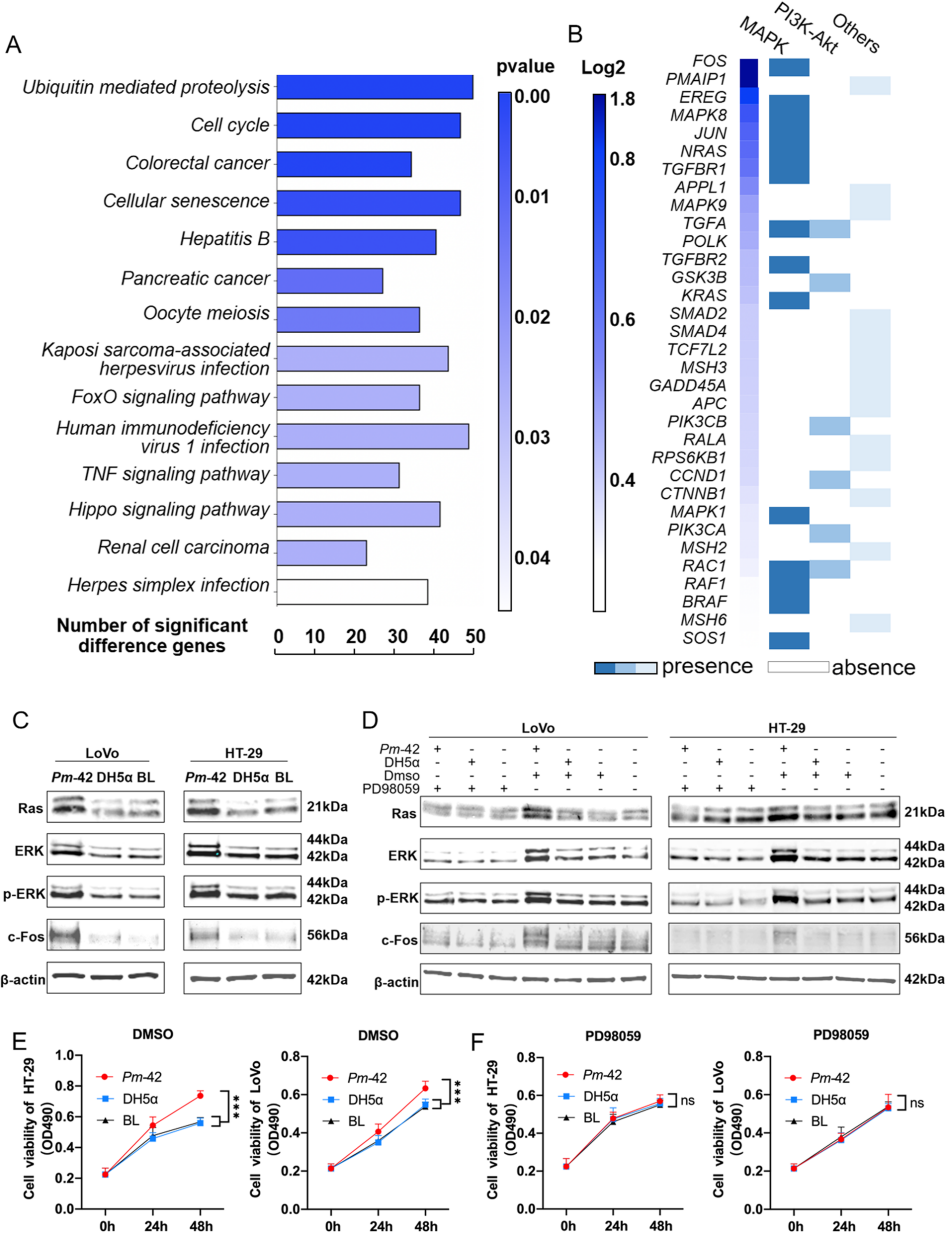
*Parvimonas micra* regulate the MAPK signaling pathway. **A** Kyoto Encyclopedia of Genes and Genomes enrichment analysis of the upregulated genes in the *Pm-*42 group. **B** Classification of upregulated genes in the *Pm-*42 group in the colorectal cancer signaling pathway. **C** After cells and bacteria were coincubated, Ras, ERK1/2, p-ERK1/2, and c-Fos were detected through western blotting. **D** Coincubation of the cells and bacteria in the presence of the PD98059 inhibitor and detection of Ras, ERK1/2, p-ERK1/2, and c-Fos through western blotting. **E**–**F** DMSO or PD98059 inhibitors were added to the coincubated cells and bacteria; the MTT assay was used to detect cell proliferation. Data are expressed as mean ± SD from 3 independent experiments

We incubated *P. micra* with cells at an MOI of 10 for 48 h, and then collected the cells for cell transcriptome sequencing analysis. Compared with the blank group, more up- and downregulated genes were present in the *Pm-*42 group (Supplemental Fig. 4A and B). Kyoto Encyclopedia of Genes and Genomes (KEGG) enrichment analyses were performed for the up- and downregulated genes (Fig. 3A and Supplemental Fig. 4C). In the up-regulated genes of *Pm-42* group compared with the blank group, 14 significant differential signal KEGG pathways were found. The top three with the most significant differences included ubiquitin-mediated proteolysis, cell cycle, and colorectal cancer signaling (Fig. 3 A). We calculated and ranked the fold change of genes with significant differences in the three pathways. Colorectal cancer signaling had the largest difference between the *Pm*-42 and blank groups (Supplemental Fig. 4D). We then classified the genes that were significantly upregulated in the colorectal cancer pathway. Among them, the most significantly upregulated genes were clustered in the mitogen-activated protein kinase (MAPK) signaling pathway (Fig. 3B). Therefore, we hypothesized that *P. micra* promotes tumor cell proliferation through the MAPK pathway. The cells and bacteria were coincubated, and the key proteins of MAPK pathway were detected. Levels of Ras, ERK1/2, p-ERK1/2, and c-Fos were significantly higher in the *Pm-*42 group than in the DH5α and blank groups (Fig. 3C and Supplemental Fig. 5A). Similarly, we also verified this in APC^Min/+^ mouse intestinal tissue, and the results were consistent in vivo and in vitro (Supplemental Fig. 5B and C). Then the ERK1/2 inhibitor PD98059 was used to inhibit the Ras/ERK/c-Fos pathway. Results showed that the difference in protein levels disappeared after inhibitor addition (Fig. 3D and Supplemental Fig. 6). Thus, *P. micra* can promote the proliferation of tumor cells through the Ras/ERK/c-Fos pathway. Finally, we verified the disappearance of cell proliferation difference in the presence of the PD98059 inhibitor using the MTT assay (Fig. 3E and F), which further indicated that *P. micra* promotes tumor cell proliferation through the activation of the Ras/ERK/c-Fos signaling pathway.

### Screening for related microRNAs of *P. micra* promoting CRC development

**Fig. 4:**
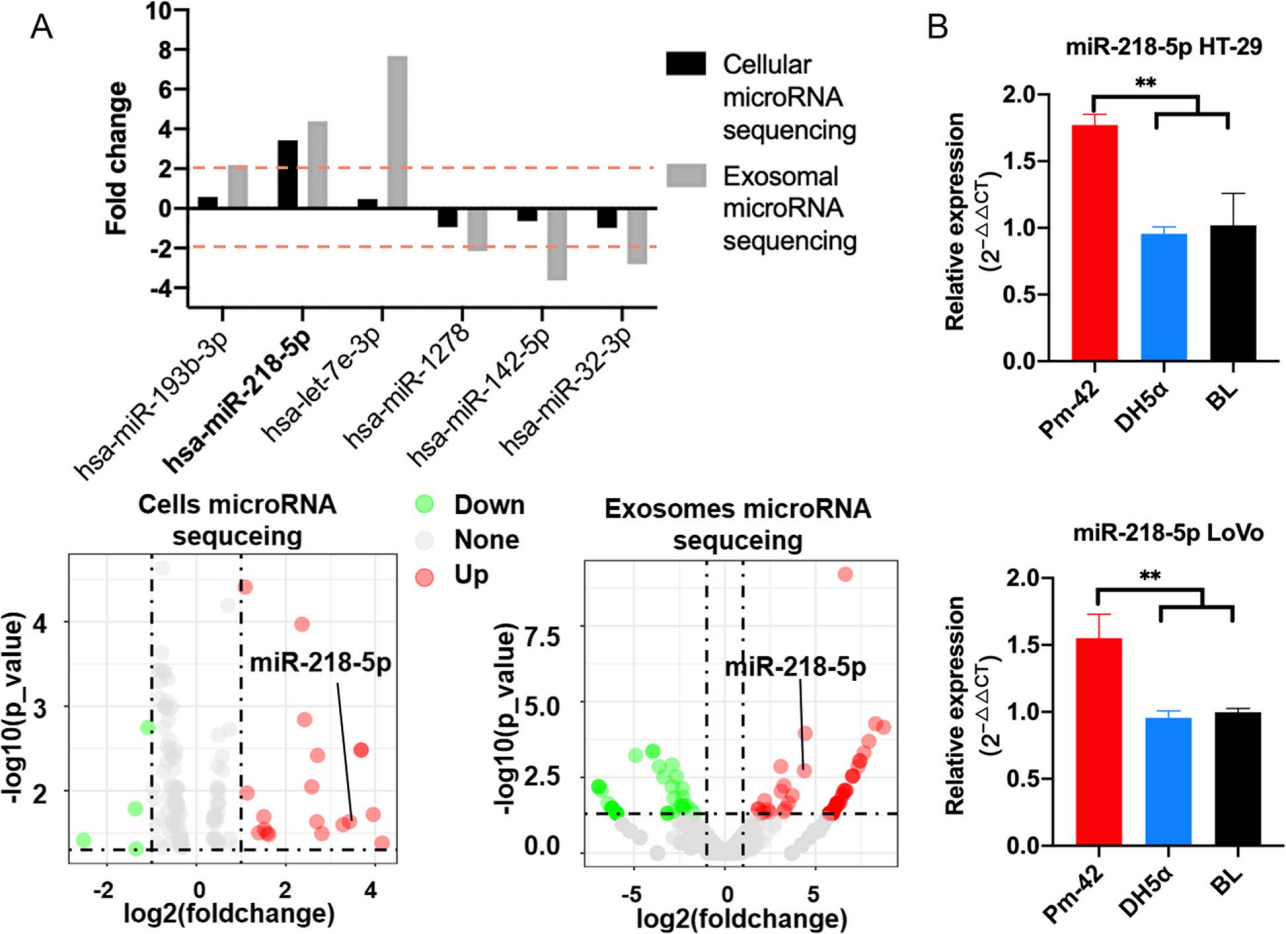
miR-218-5p is upregulated in both cellular and exosomal fractions. **A** Compared to the blank group, six microRNAs were up- or downregulated in both the cells and exosomes of the *Pm*-42 group. **B** qPCR detection of miR-218-5p in the cells after coincubation with *Pm-*42, DH5α, or PBS. Data are expressed as mean ± SD from 3 independent experiments

MicroRNA is an important factor in the development of cancer, and plays an important role in the regulation of signaling pathways. Therefore, we performed cellular and exosomal microRNA sequencing analysis after the coincubation of *P. micra* and cells. There were significant differences in Colorectal cancer, Pathways in cancer and PI3K-Akt signaling pathway in KEGG pathway enrichment analysis of differential microRNA target genes, indicating that differential microRNA expression and CRC may be closely linked (Supplemental Fig. 7). Cellular and exosomal microRNA analysis revealed the presence of hundreds of microRNAs significantly up- or downregulated between the *Pm*-42 and blank groups (Supplemental Tables 3 and 4); however, only six microRNAs were significantly up- or downregulated in both cellular and exosomal microRNA sequencing (Fig. 4A). We analyzed the six microRNAs and screened out microRNAs with |log2| greater than two in both cellular and exosomal sequencing. Finally, one microRNA, miR-218-5p, was found to be significantly upregulated in both the cells and exosomes (Fig. 4A). We used qPCR to detect the abundance of miR-218-5p after coincubation of bacteria and cells. The results showed that miR-218-5p levels in the *Pm-*42 group were significantly higher than that in the DH5α and blank groups (Fig. 4B).

### *P. micra* regulates the expression of miR-218-5p and its target gene *PTPRR*

**Fig. 5:**
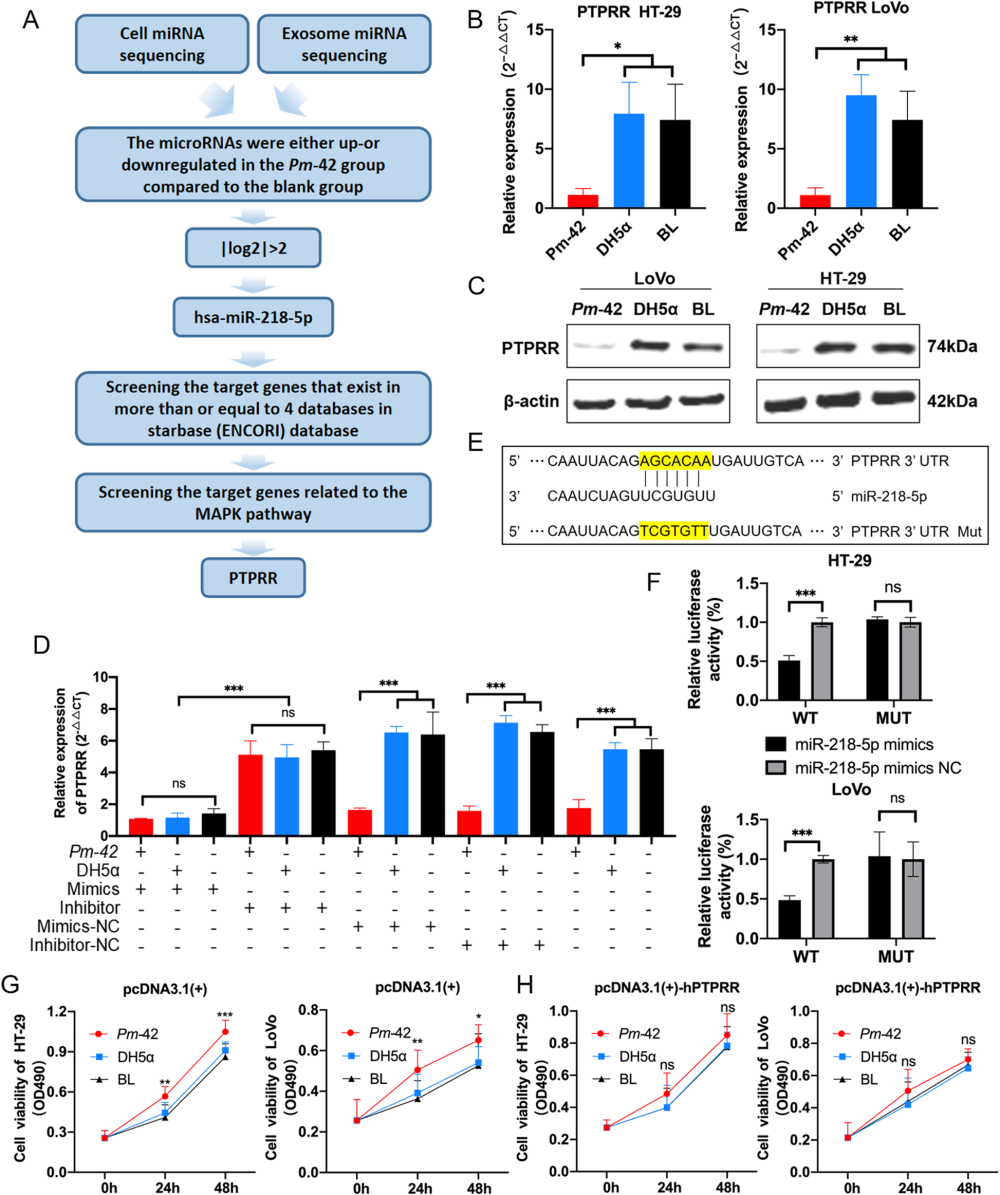
Protein tyrosine phosphatase receptor R (PTPRR) is a target of miR-218-5p. **A** A schematic diagram for screening *Parvimonas micra*-regulated microRNAs and target genes. **B** qPCR detection of the PTPRR in the cells after coincubation with *Pm-*42, DH5α, or PBS. **C** Western blot detection of PTPRR in the cells after coincubation with *Pm-*42, DH5α, or PBS. **D** The mimics, inhibitor, or negative control (NC) of miR-218-5p were transfected into LoVo cells and coincubated with *Pm-*42, DH5α, or PBS. Then *PTPRR* was detected through qPCR. **E** The binding sites of miR-218-5p and PTPRR were predicted using TargetScan; dual luciferase plasmids and mutants containing PTPRR binding sequences were constructed. **F** miR-218-5p binding to *PTPRR* was detected using the luciferase reporter gene assay. **G**–**H** pcDNA3.1(+)-hPTPRR or pcDNA3.1(+) vectors were transfected into LoVo or HT-29 cells, then coincubated with bacteria; the MTT assay was used to detect cell proliferation. Data are expressed as mean ± SD from 3 independent experiments

We confirmed that the Ras/ERK/c-Fos signal pathway plays an important role in *P. micra* promoting CRC development. Moreover, microRNA sequencing results revealed that miR-218-5p was highly expressed both in cells and exosomes. Using starBase, a database which contains seven micorRNA target genes, we looked for target genes of miR-218-5p. First, we determined the target genes present in ≥4 databases in the starBase (ENCORI) database, and then screened target genes related to the MAPK signal pathway. We finally screened out the protein tyrosine phosphatase receptor type R (*PTPRR*) (Fig. 5A). PTPRR can dephosphorylate p-ERK, thus inhibiting the MAPK pathway [28]. We used qPCR to detect the abundance of *PTPRR* after coincubation of bacteria and cells and found that *PTPRR* level was significantly lower in the *Pm-*42 group than that in the DH5α and blank groups (Fig. 5B). At the protein level, PTPRR expression in the *Pm-*42 group was significantly lower than that in the DH5α or blank groups (Fig. 5C and Supplemental Fig. 8A). We used qPCR and the luciferase reporter gene assay to verify the relationship between miR-218-5p and PTPRR. After transfecting with mimicss or inhibitors, the differences between *Pm-*42, DH5α, and the blank group disappeared, whereas PTPRR expression was significantly higher in cells transfected with inhibitors compared with transfected mimics (Fig. 5D and Supplemental Fig. 8B). These results indicated a negative correlation between miR-218-5p and PTPRR. The presence of difference in the negative control group indicates that the negative control group of inhibitors and mimics has no effect on the target gene. The binding sites of miR-218-5p and PTPRR were predicted using TargetScan, and dual luciferase plasmids and mutants containing PTPRR binding sequences were constructed (Fig. 5E). The miR-218-5p mimics (or miR-218-5p mimics negative control) and plasmid were cotransfected into HT-29 and LoVo cells to observe the interaction. The detection value of the wild type plasmid was significantly lower than that of the negative control group, whereas the mutation group showed no significant differences (Fig. 5F), which further indicated that an interactive relationship between miR-218-5p and PTPRR. Finally, we transfected the plasmid (pcDNA3.1(+)-hPTPRR) highly expressing the PTPRR protein in the cell lines, and then infected the cells with the *Pm-42*, DH5α strains to examine the cell proliferation and mRNA expression of the Ras/ERK/c-Fos signaling pathway. The results showed that the differences in cell proliferation (Fig. 5G and H) and mRNA expression (Supplemental Fig. 8C) among the three groups disappeared, indicating the important role of PTPRR protein in activating the Ras/ERK/c-Fos signaling pathway to promote cell proliferation mechanism by *P. micra*.

### The role of miR-218-5p in the development of CRC is promoted by *P. micra*

**Fig. 6:**
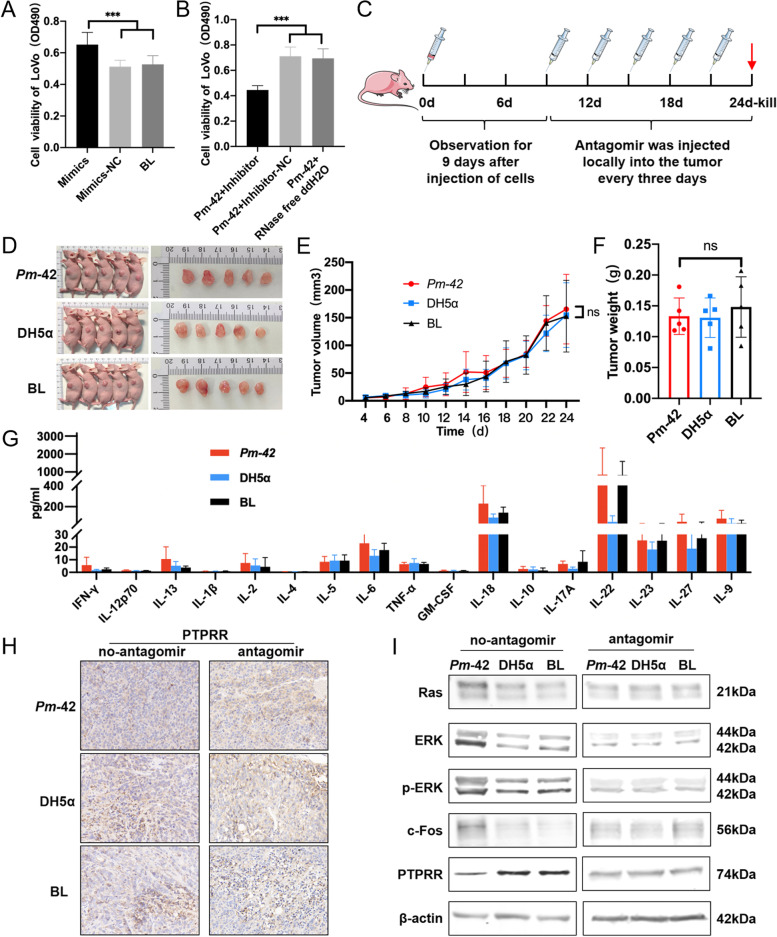
miR-218-5p inhibitor recovered phenotypes of mice induced by *Parvimonas micra. ***A** The mimics, inhibitor, or negative control (NC) of miR-218-5p was transfected into LoVo cells, and the MTT assay was used to detect cell proliferation. **B** The miR-218-5p inhibitor, miR-218-5p inhibitor negative control, or RNase free ddH_2_O was added into LoVo cells and then coincubated with *Pm-42*, and the MTT assay was used to detect cell proliferation. Data are expressed as mean ± SD from 3 independent experiments. **C** LoVo or HT-29 cells treated with *Pm-42*, DH5α, or PBS were subcutaneously injected into male BALB/C nude mice to produce xenograft tumors in animals. After nine days of cell injection, the antagomir was injected locally into the tumor every three days for a total of five injections. **D**–**G** LoVo cells treated with *Pm-42*, DH5α, or PBS were subcutaneously injected into male BALB/C nude mice to produce xenograft tumors in animals. Data are expressed as mean ± SD, n = 5. **D** Images of xenograft mice. **E** The change of tumor size in xenograft mice. **F** The weight of tumor in xenograft mice. **G** The detection of 17 cytokines in the serum of xenograft mice. **H** Immunohistochemistry was used to detect the PTPRR levels in xenograft mice of LoVo cells. **I** Western blotting was used to detect Ras, ERK1/2, p-ERK1/2, c-Fos and PTPRR in the xenograft mice of LoVo cells. Data are expressed as mean ± SD from 3 independent experiments

To further verify the role of miR-218-5p in the promotion of CRC development by *P. micra*, we transfected the cell lines and animal models with the miR-218-5p inhibitor. Results from the MTT assay in LoVo and HT-29 cells showed that the addition of miR-218-5p mimics alone significantly promoted cell proliferation compared with the negative control and blank groups (Fig. 6 A and Supplemental Fig. 9A). Simultaneously, the use of the miR-218-5p inhibitor could significantly block the proliferation effect induced by *Pm-*42 (Fig. 6B and Supplemental Fig. 9B). The in vitro results showed that miR-218-5p was an important factor for *P. micra* in promoting cell proliferation. Next, we performed the xenograft model experiment in nude mice. We injected 6-week-old female nude mice subcutaneously with LoVo or HT-29 cells infected with *Pm-*42, DH5α, or PBS. The tumor site was locally injected with the miR-218-5p inhibitor (antagomir) nine days after inoculation, once every three days for a total of five injections (Fig. 6C). The size and weight of the tumor were noted. The results revealed that after the injection of the antagomir, the difference between *Pm-*42 and the other two groups (DH-5α and blank) disappeared, whether in gross observation, the tumor size and tumor weight (Figs. 6D-F and Supplemental Fig. 9C). In addition, we detected no significant differences in the 17 cytokines evaluated in mice sera between the three groups (Fig. 6G). IHC detection of ERK1/2, c-Fos, and PTPRR was performed on the tumor tissues of the two xenograft model experiments. Without the antagomir, ERK1/2 and c-Fos expression in the *Pm-*42 group was significantly higher than that in the DH5α and blank groups, and PTPRR was lower than the two groups, whereas the difference disappeared after using the antagomir (Fig. 6H, Supplemental Fig. 9D-F). Similar to IHC results, western blot analysis revealed that levels of Ras, ERK1/2, p-ERK1/2, and c-Fos significantly increased, whereas that of PTPRR significantly decreased in the *Pm-*42 group. However, the difference disappeared when the mice were injected with the antagomir (Fig. 6I and Supplemental Fig. 10A-C). These results suggest that *P. micra* promotes the development of CRC by upregulating the transcription of miR-218-5p, inhibiting the expression of *PTPRR*, and then activating the Ras/ERK/c-Fos signaling pathway.

### In CRC tissues, the high expression of miR-218-5p is associated with a high abundance of *P. micra*, indicating poor clinical prognosis

**Fig. 7:**
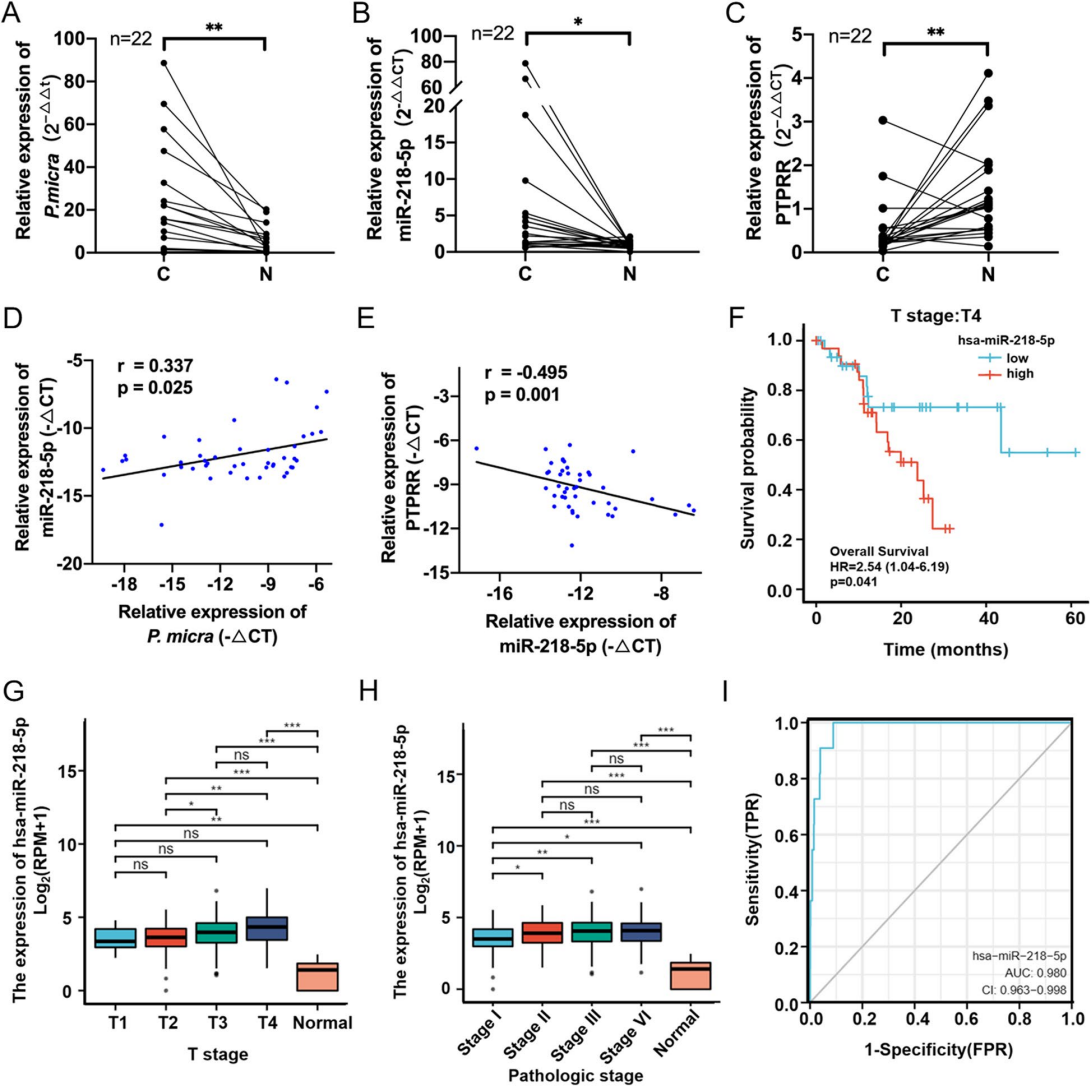
Analysis of clinical data indicates the potential diagnostic role of miR-218-5p in colorectal cancer. **A**–**C** Comparison of miR-218-5p, PTPRR expression, and *Parvimonas micra* abundance in cancer mucosa (**C**) and adjacent normal tissues (**N**). **D**–**E** Correlation analysis between *P. micra* abundance and miR-218-5p or miR-218-5p and PTPRR expression. **F** Survival rates of high- and low-expressing miR-218-5p in stage T4 samples from the TCGA data. **G** Expression of miR-218-5p in stages T1–T4 in the TCGA data. **H** Expression of miR-218-5p in pathological stages I–IV in the TCGA data. **I** ROC curve for the diagnosis of colorectal cancer based on miR-218-5p

To further verify our conclusions from clinical data, we collected 44 samples of CRC mucosa and adjacent normal tissues from 22 patients with CRC, and detected an abundance of *P. micra*, miR-218-5p, and PTPRR expression using qPCR. The abundance of *P. micra* in cancer tissue was significantly higher than that in adjacent normal tissue (Fig. 7A). Moreover, the expression of miR-218-5p was significantly increased (Fig. 7B), whereas that of *PTPRR* was decreased (Fig. 7C). These results indicate an important role between *P. micra*, miR-218-5p, and PTPRR in the development of CRC. We further evaluated the association between *P. micra* and miR-218-5p and miR-218-5p and PTPRR. Correlation analysis revealed a significant positive correlation between *P. micra* and miR-218-5p (Fig. 7D) and a significant negative correlation between miR-218-5 and PTPRR (Fig. 7E). We analyzed the clinical outcomes associated with miR-218-5p and PTPRR using another database from TCGA and found significant differences between miR-218-5p and PTPRR in both cancer mucosa and adjacent normal tissues of pan-cancer (Supplemental Fig. 11A and B). Regarding CRC data, the differences in the abundance of miR-218-5p and PTPRR in both cancer tissues and adjacent normal tissues (Supplemental Fig. 11C and D) were consistent with our data. The survival rates of patients with high miR-218-5p in clinical TNM stage T4 were significantly reduced (Fig. 7F), and the expression of miR-218-5p in patients with T1–T4 staging showed a significantly increasing trend (Fig. 7G). We also evaluated the expression of miR-218-5p and PTPRR in patients with pathological stages I–IV. The expression of miR-218-5p in patients with pathologic stages II–IV was significantly higher than that in patients with pathologic stage I (Fig. 7H), whereas the expression of PTPRR was not significantly different between pathologic stages I–IV (Supplemental Fig. 11E). In addition, the area under receiver operating characteristic (ROC) curve was 98% for miR-218-5p (Fig. 7I) and 84% for PTPRR (Supplemental Fig. 11F), indicating that miR-218-5p and PTPRR may be used as a target for the clinical detection of CRC. The information and description statistics of the samples collected in this experiment and those collected from public databases are shown in Supplemental Table 5. Thus, these clinical data show that miR-218-5p plays an important role in the development of *P. micra*-induced CRC.

## Discussion

CRC is one of the most common tumors in the world. Several studies have shown that the gut microbiota is closely related to CRC. Earlier studies have shown that the transplantation of feces from patients with CRC into germ-free mice can promote the development of CRC in mice, thus confirming that carcinogenic bacteria may exist in the flora of patients with CRC [29]. In recent years, some bacteria have been confirmed to be associated with CRC, such as ST11 *Streptococcus pneumoniae*, which can promote the development of CRC by changing the immune microenvironment [26]. *Porphyromonas gingivalis* promotes CRC by activating the NLRP3 inflammasome [8].

*P. micra* is an oral opportunistic pathogen that is closely related to periodontitis and other diseases; moreover, it can cause purulent infections of systemic organs [30, 31]. Bioinformatics analysis showed that the abundance of *P. micra* in patients with CRC was significantly higher than that in healthy people [32, 33]. Further studies evaluated the abundance of *P. micra* from the perspective of the development of polyps, adenocarcinomas, and other cancers, and speculated that it played an important role in promoting the development of cancer [34]. Thus, a close relationship exists between *P. micra* and CRC; however, experimental studies on *P. micra* are scarce.

Although many bacteria are associated with various diseases, most strains used are not isolated from the disease samples but purchased from the American type culture collection (ATCC) [35, 36], which may not be from disease samples. For *P. micra*, the ATCC strain of *P. micra* was derived from suppurative pleurisy (https://www.atcc.org/products/33270) and not from patients with CRC. In addition, previous studies have confirmed that the composition of flora in the mucosa and feces is substantially different, and mucosal flora affects the occurrence and development of diseases more [37, 38]. Here we isolated a strain of *P. micra* from the mucosal tissues of patients with CRC. This strain was used for all experiments, making the experimental results more credible.

We first confirmed that *P. micra* can promote the development of CRC both in vivo and in vitro. In the gavage experiment with APC^Min/+^ mice, the levels of LDH, ALP, and Cr were significantly higher in *Pm-*42-administrated mice. An increase in LDH levels common in malignant tumors, myocardial infarction, and liver disease [39]. High ALP levels are commonly seen in liver disease, bone metastasis of malignant tumors, etc. [40]. Elevated creatinine is common in diseases such as kidney disease [41]. An increase in these indexes further proved that the tumors in the *Pm-*42 group were more malignant. Studies have shown that IL-1β, TNF-α, and IL-6 are the most substantially changed cytokines in cancer, and their levels can even be related to cancer stage [42, 43]. Therefore, the significant increase of serum TNF-α and IL-6 in the *Pm-*42 group at both six and 12 weeks confirms that *Pm-*42 has a stronger proinflammatory effect. Thus, *P. micra* can contribute to the development of CRC.

MicroRNAs play an important role in CRC development, studies have shown that microRNAs plays a regulatory role in CRC related autophagy [44]. In addition, various cancer-associated bacteria can promote CRC development by regulating microRNAs. Bacteria such as *Fusobacterium nucleatum*, which causes CRC, promote the development of CRC by activating toll-like receptor 4 and upregulating microRNA-21 expression [18]. Toxigenic *Bacteroides fragilis* relies on METTL14-mediated m6A methylation to downregulate miR-149-3p, and thereby, promote CRC proliferation [19]. In this study, we demonstrated that *P. micra* can promote the development of CRC by upregulating the expression of miR-218-5p. Although miR-218-5p has been scarcely investigated in CRC studies, miR-218-5p was shown to directly target *SFRP2* to upregulate the WNT/β-catenin pathway in a study on promoting hair proliferation [45, 46]. In addition, the miR-218/WNT signaling pathway was found to be activated in the bone metastasis of breast cancer cells [47].

**Fig. 8:**
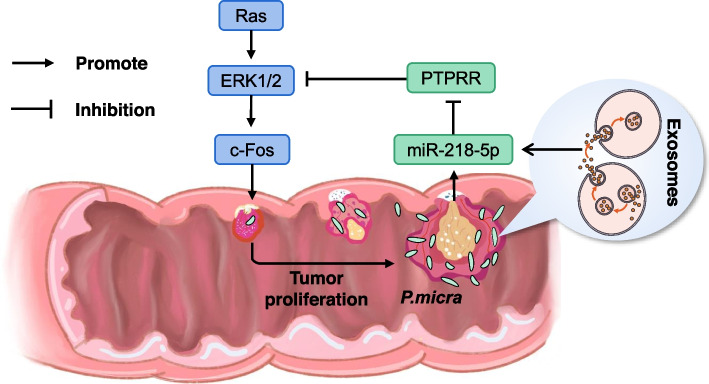
Schematic diagram of the development and pathogenic mechanism of *Parvimonas micra* in colorectal cancer

To identify target genes regulated by miR-218-5p, we compared the starBase (ENCORI) database and confirmed the presence of PTPRR. Protein tyrosine phosphatase regulates various cellular processes, including cell growth, differentiation, mitosis, and oncogenic transformation [48]. *PTPRR* significantly inhibits the activation of the MAPK cascade [28]. As the most important signaling cascade in all MAPK signal transduction pathways, the Ras/ERK/c-Fos pathway plays a crucial role in the survival and development of tumor cells [49]. However, studies on the relationship between bacteria and MAPK signaling pathway in CRC are lacking. Here we confirmed that *P. micra* could promote CRC through the upregulation of miR-218-5p, which leads to the downregulation of PTPRR and then the activation of the MAPK signaling pathway (Fig. 8). Moreover, *P. micra* is an upstream activator of the miR-218-5p/PTPRR/MAPK signaling pathway.

To verify the clinical applicability of these data, we collected samples from patients with CRC and also analyzed data from the TCGA database. Notably, according to stage T in TNM, miR-218-5p expression in T4 was significantly higher than that in T1–T3, and the survival rates of patients with high miR-218-5p expression in T4 were significantly lower. In the TNM staging system, T (tumor) stage refers to the condition of the primary tumor, and a progressive increase in tumor volume and the extent of adjacent tissue involved is represented by T1–T4, meanwhile, some studies have suggested that T stage has greater weight than N stage in the staging of CRC [50]. Therefore, miR-218-5p may be closely related to primary tumors. The expression of miR-218-5p was significantly higher in the middle and late pathological stages II–IV than in early stages, indicating that miR-218-5p plays an important role in disease development. Moreover, the increase in miR-218-5p has a direct relationship with *P. micra*. In summary, miR-218-5p and *P. micra* may be new targets for the prevention, diagnosis, and treatment of CRC.

The relationship between gut microbiota and CRC is complex and has not been characterized or studied extensively. Therefore, identifying harmful intestinal microbiota in patients is of great significance. The purpose of our study was to identify biomarkers as indicators of early diagnosis and prognosis and apply bacterial targeted therapy of rectal cancer for treating or preventing CRC. In the future, it is important to study the relationship between gut microbiota and CRC by isolating patient-derived strains through culturomics, combining this data with metagenomic data, and evaluate disease pathogenesis.

## Supplementary Information

**Additional file 1.** 

**Additional file 2.**

**Additional file 3.**

**Additional file 4.**

**Additional file 5.**

**Additional file 6.**

## Data Availability

All the data generated or analyzed in this study are included in this published article (or its Supplementary Information files).

## Abbreviations

CRC: Colorectal cancer
*P.micra*: *Parvimonas micra*
PTPRR: Protein tyrosine phosphatase receptor R
MOI: Multiplicity of infection
RTCA: Real-time cell analysis
IHC: Immunohistochemical
SDS-PAGE: Sodium dodecyl sulfate polyacrylamide gel electrophoresis
qPCR: Quantitative polymerase chain reaction
ANOVA: Analysis of variance
LDH: Lactate dehydrogenase
Cr: Creatinine
ALP: Alkaline phosphatase
PCoA: Principal coordinate analysis
LEfSe: Linear discriminant analysis effect size
KEGG: Kyoto Encyclopedia of Genes and Genomes
ROC: Receiver operating characteristic
ATCC: American type culture collection
