# Supplementary material for: Parvimonas micra activates the Ras/ERK/c-Fos pathway by upregulating miR-218-5p to promote colorectal cancer progression
Source: J Exp Clin Cancer Res. 2023 Jan 10;42:13. doi: 10.1186/s13046-022-02572-2 (PMC9830783; doi:10.1186/s13046-022-02572-2)
Supplement: Supplementary file 1 — Additional file 1. [file 13046_2022_2572_MOESM1_ESM.pptx]

## Slide 1
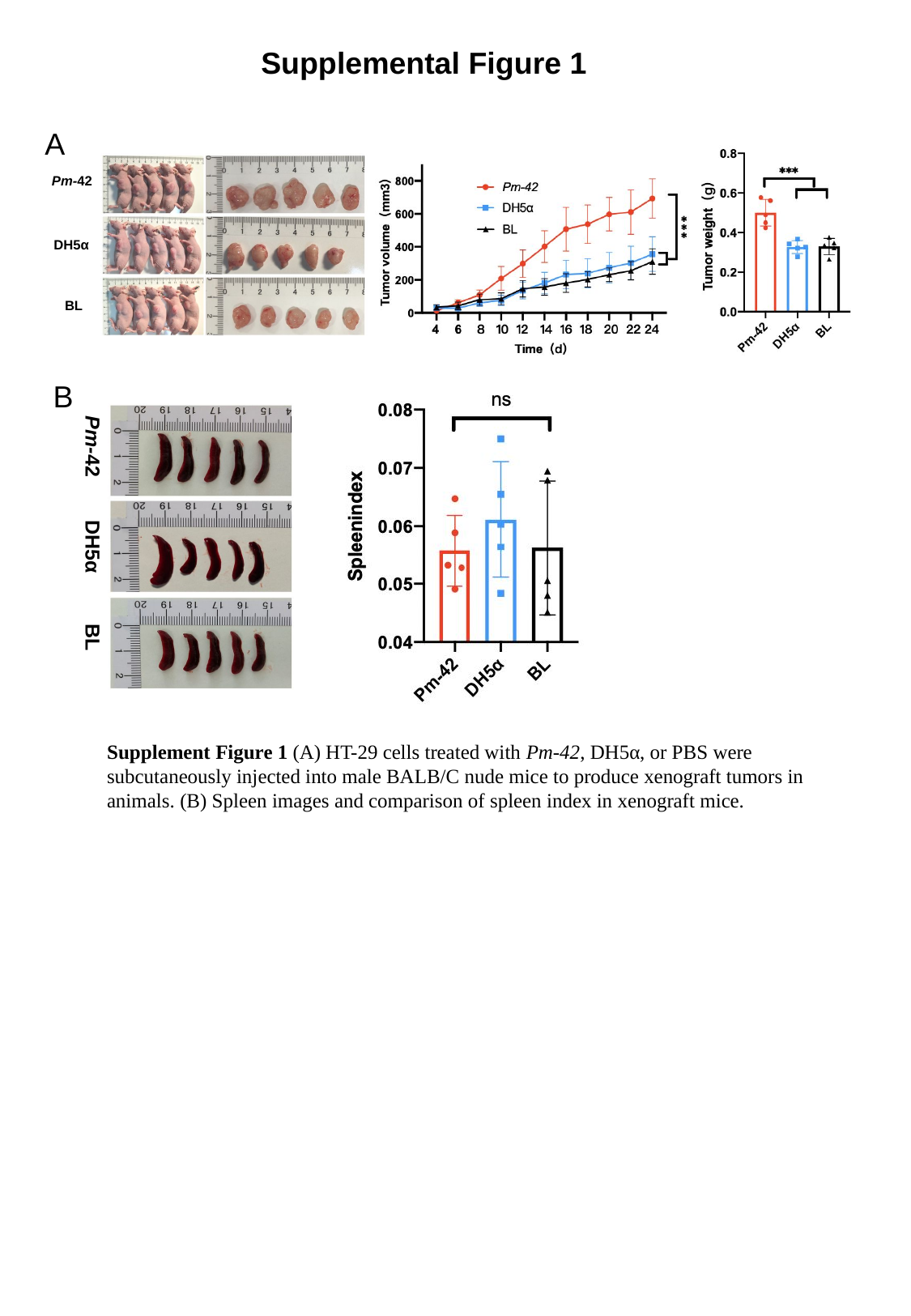

Supplemental Figure 1
A
Pm-42
DH5α
BL
B
Pm-42
DH5α
BL
Supplement Figure 1 (A) HT-29 cells treated with Pm-42, DH5α, or PBS were subcutaneously injected into male BALB/C nude mice to produce xenograft tumors in animals. (B) Spleen images and comparison of spleen index in xenograft mice.

## Slide 2
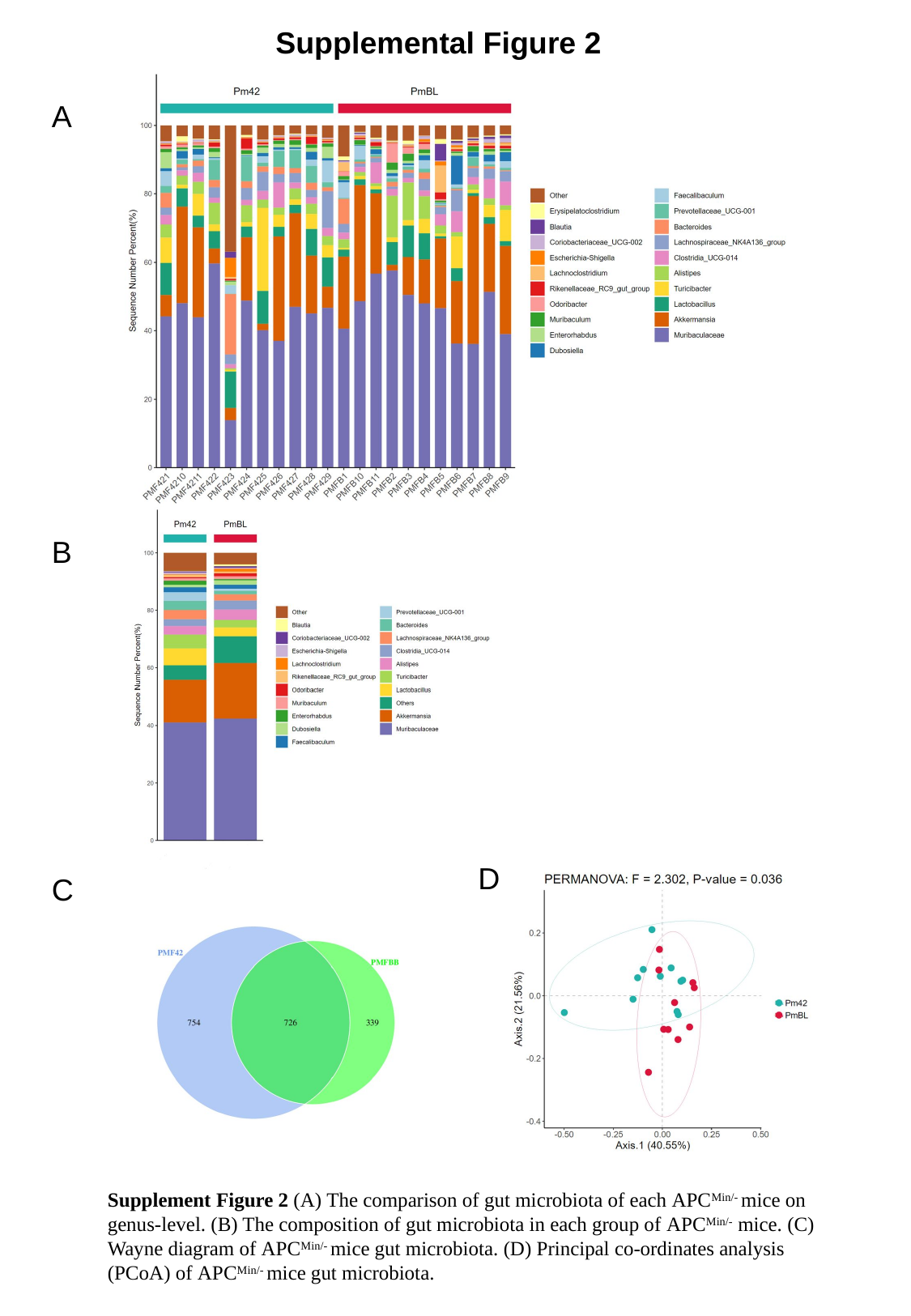

Supplemental Figure 2
A
B
D
C
Supplement Figure 2 (A) The comparison of gut microbiota of each APCMin/- mice on genus-level. (B) The composition of gut microbiota in each group of APCMin/- mice. (C) Wayne diagram of APCMin/- mice gut microbiota. (D) Principal co-ordinates analysis (PCoA) of APCMin/- mice gut microbiota.

## Slide 3
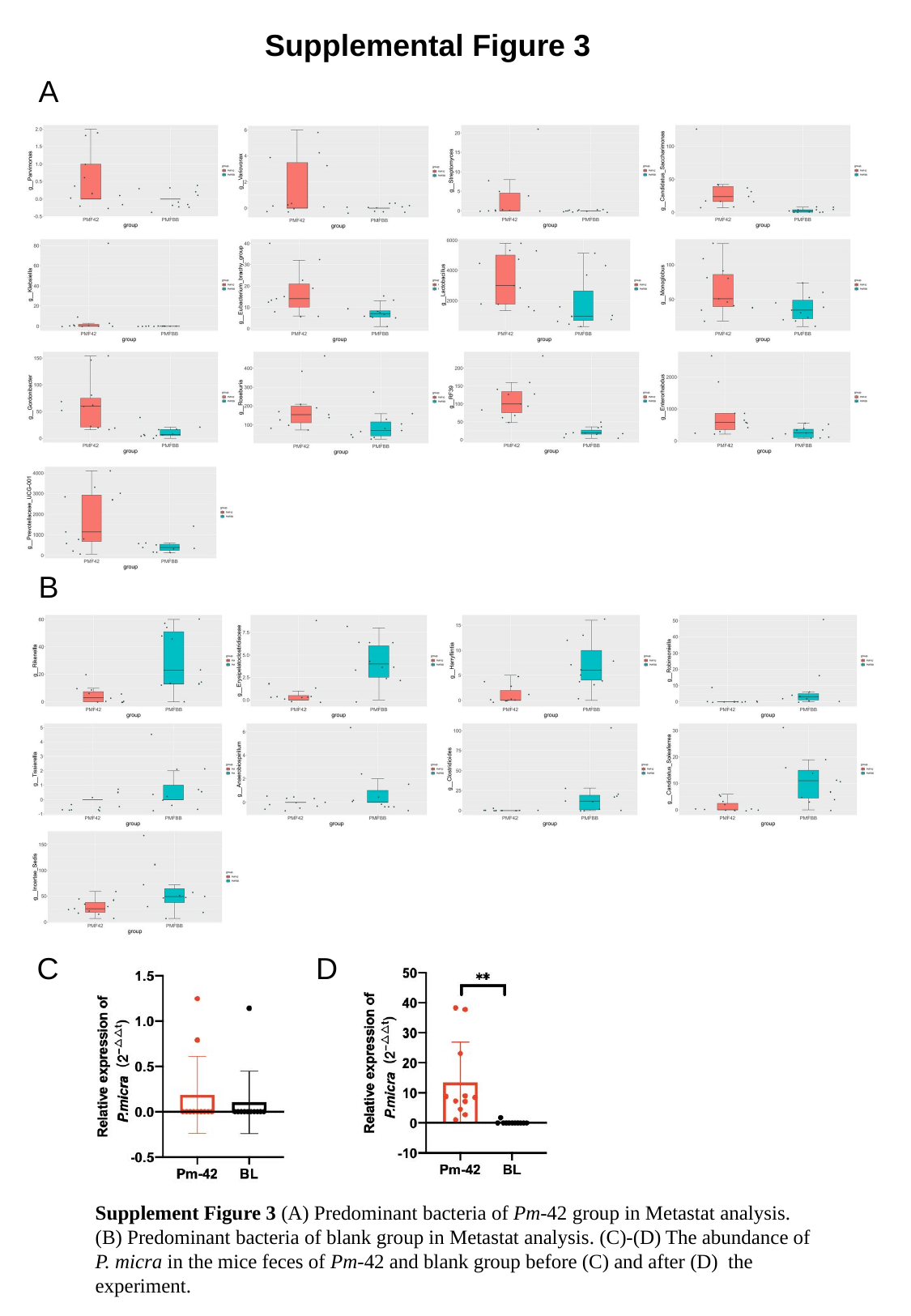

Supplemental Figure 3
A
B
C
D
Supplement Figure 3 (A) Predominant bacteria of Pm-42 group in Metastat analysis. (B) Predominant bacteria of blank group in Metastat analysis. (C)-(D) The abundance of P. micra in the mice feces of Pm-42 and blank group before (C) and after (D) the experiment.

## Slide 4
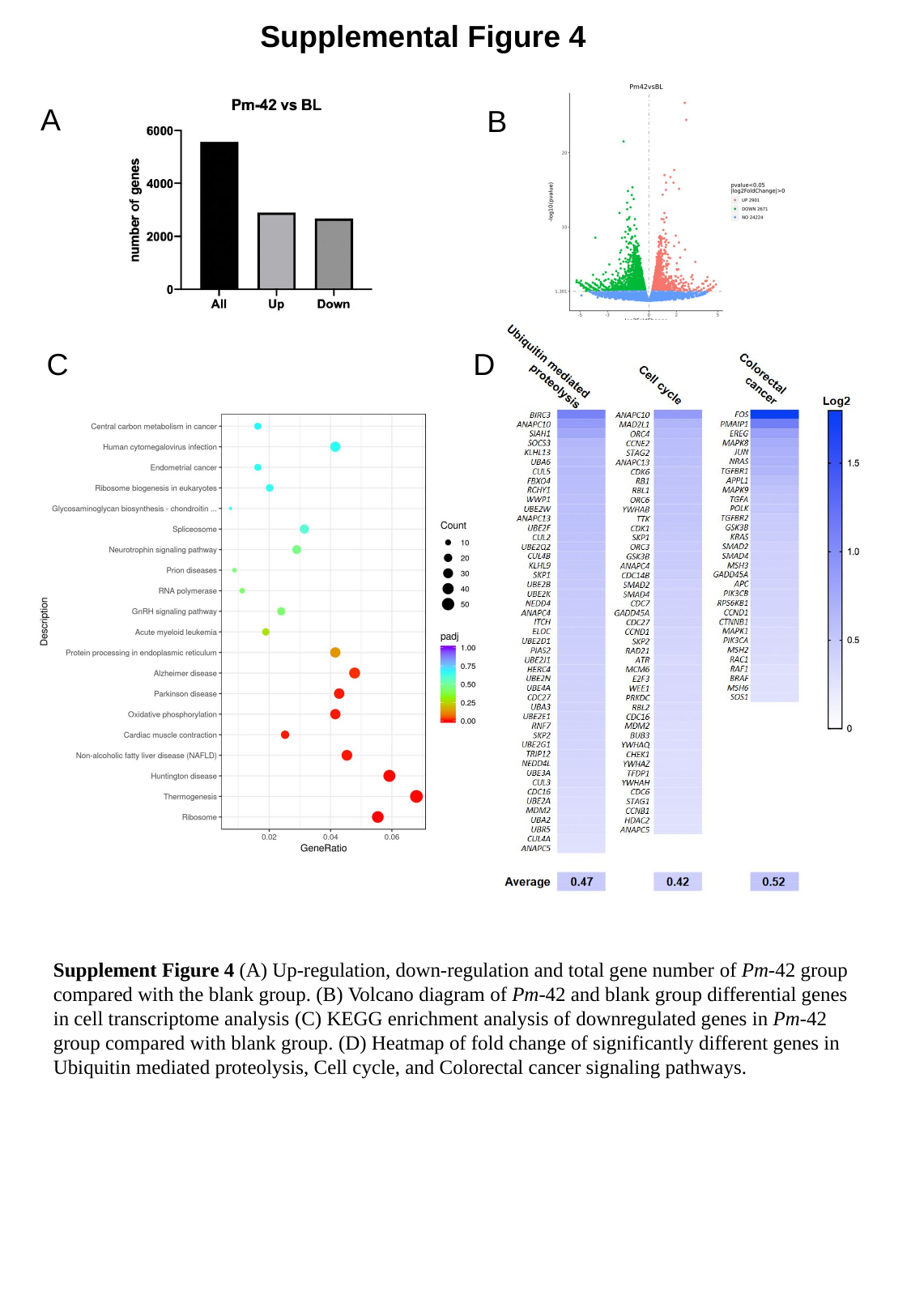

Supplemental Figure 4
A
B
C
D
Supplement Figure 4 (A) Up-regulation, down-regulation and total gene number of Pm-42 group compared with the blank group. (B) Volcano diagram of Pm-42 and blank group differential genes in cell transcriptome analysis (C) KEGG enrichment analysis of downregulated genes in Pm-42 group compared with blank group. (D) Heatmap of fold change of significantly different genes in Ubiquitin mediated proteolysis, Cell cycle, and Colorectal cancer signaling pathways.

## Slide 5
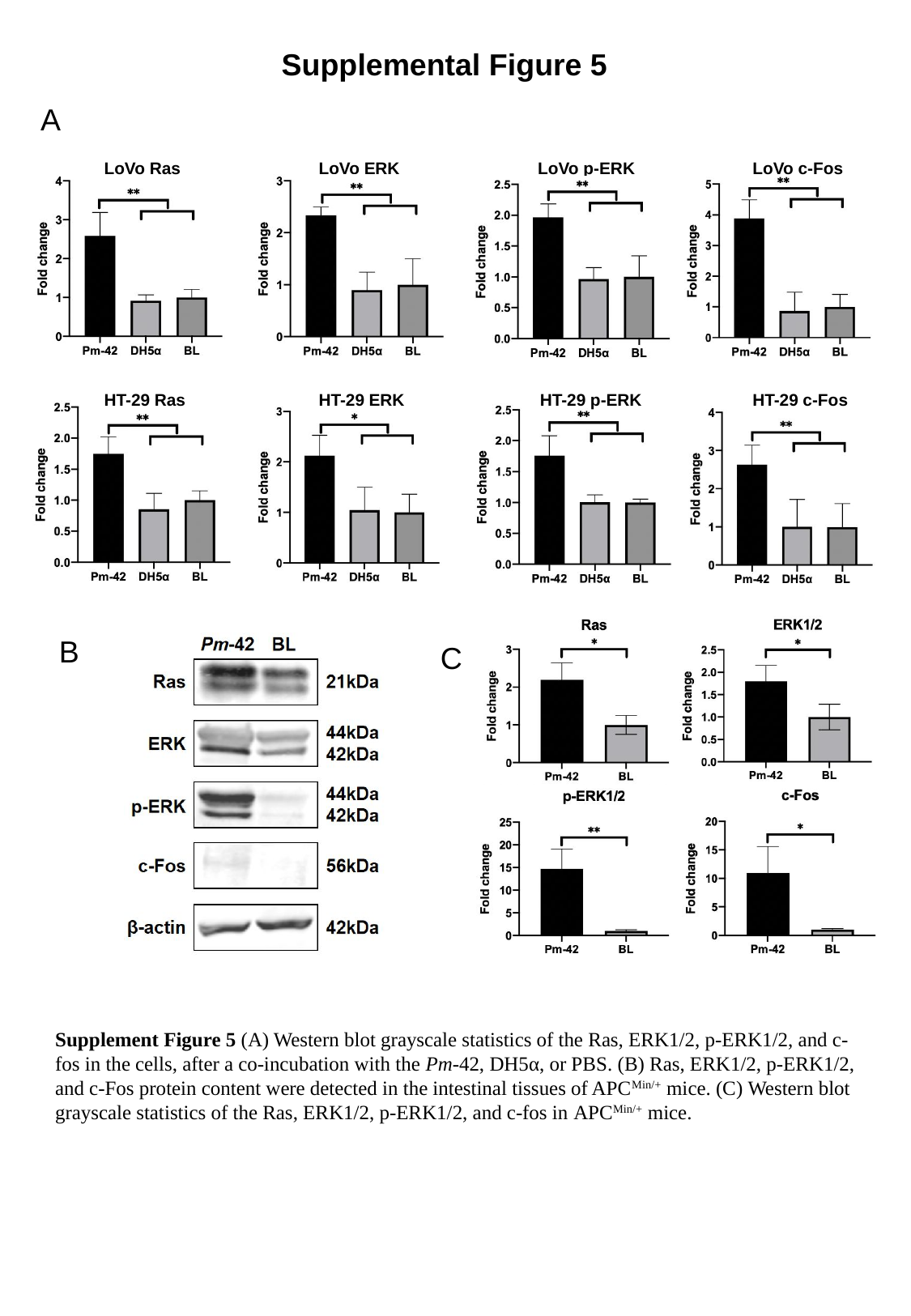

Supplemental Figure 5
A
LoVo Ras
LoVo ERK
LoVo p-ERK
LoVo c-Fos
HT-29 Ras
HT-29 ERK
HT-29 p-ERK
HT-29 c-Fos
B
C
Supplement Figure 5 (A) Western blot grayscale statistics of the Ras, ERK1/2, p-ERK1/2, and c-fos in the cells, after a co-incubation with the Pm-42, DH5α, or PBS. (B) Ras, ERK1/2, p-ERK1/2, and c-Fos protein content were detected in the intestinal tissues of APCMin/+ mice. (C) Western blot grayscale statistics of the Ras, ERK1/2, p-ERK1/2, and c-fos in APCMin/+ mice.

## Slide 6
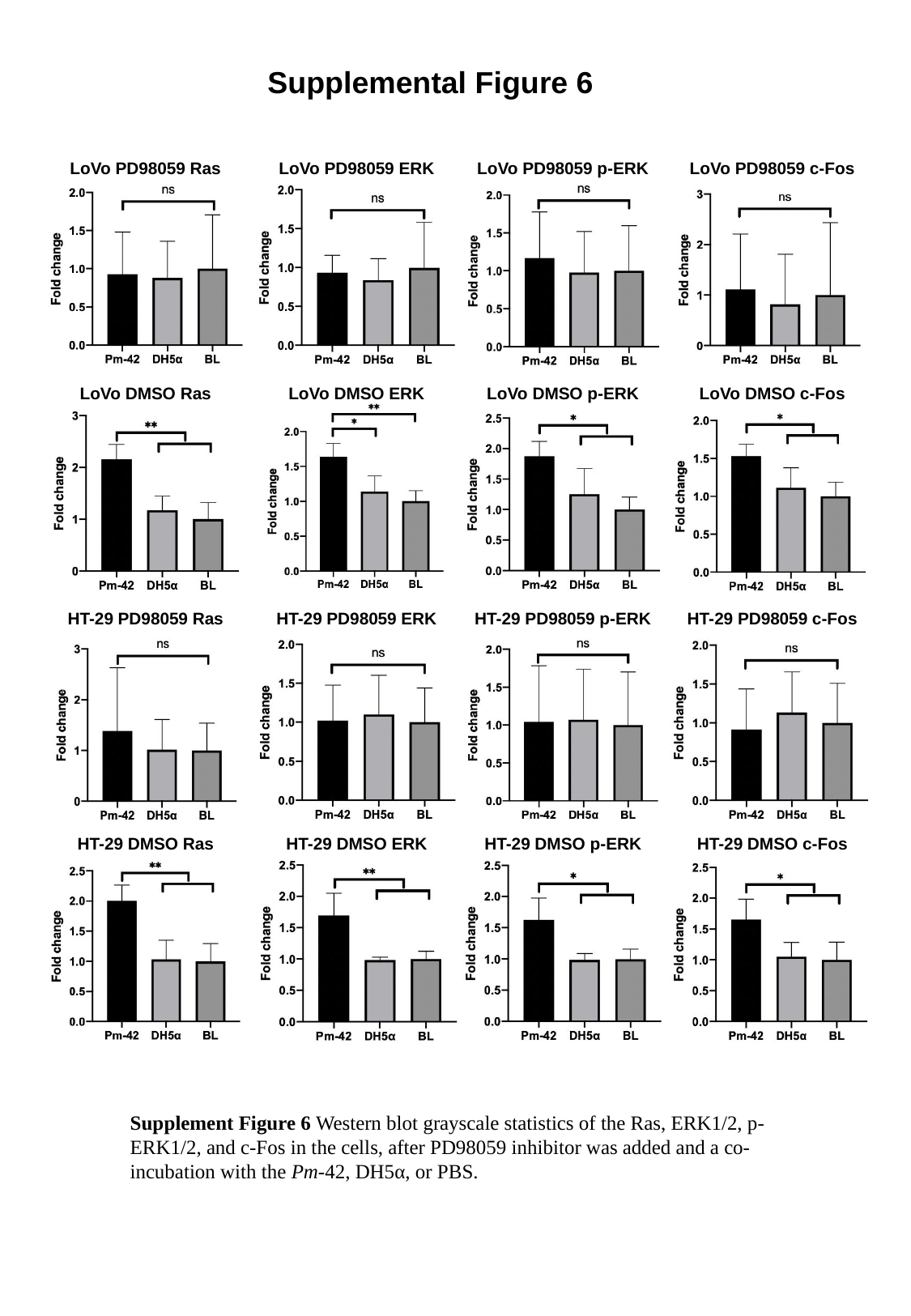

Supplemental Figure 6
LoVo PD98059 Ras
LoVo PD98059 ERK
LoVo PD98059 p-ERK
LoVo PD98059 c-Fos
LoVo DMSO Ras
LoVo DMSO ERK
LoVo DMSO p-ERK
LoVo DMSO c-Fos
HT-29 PD98059 Ras
HT-29 PD98059 ERK
HT-29 PD98059 p-ERK
HT-29 PD98059 c-Fos
HT-29 DMSO Ras
HT-29 DMSO ERK
HT-29 DMSO p-ERK
HT-29 DMSO c-Fos
Supplement Figure 6 Western blot grayscale statistics of the Ras, ERK1/2, p-ERK1/2, and c-Fos in the cells, after PD98059 inhibitor was added and a co-incubation with the Pm-42, DH5α, or PBS.

## Slide 7
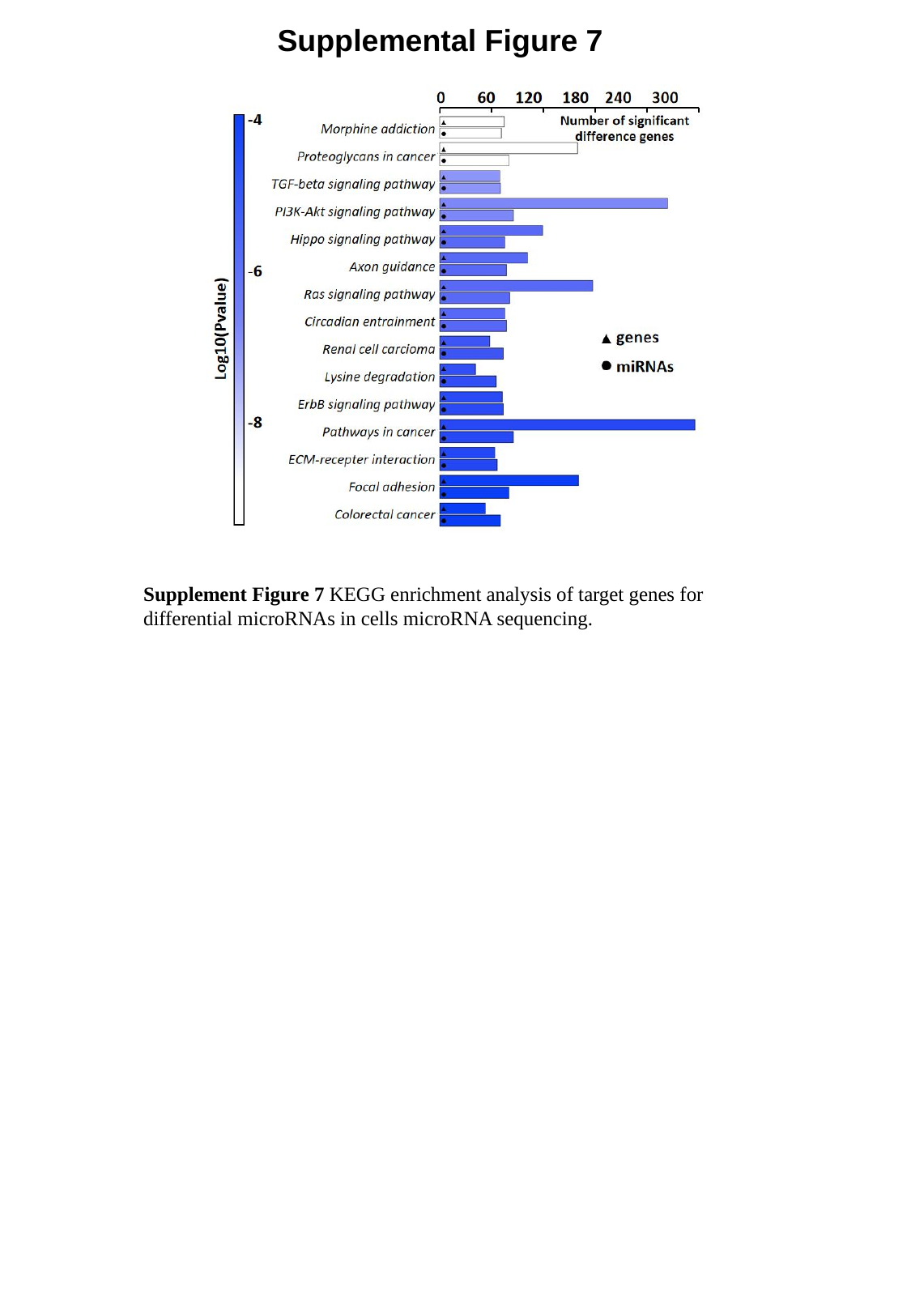

Supplemental Figure 7
Supplement Figure 7 KEGG enrichment analysis of target genes for differential microRNAs in cells microRNA sequencing.

## Slide 8
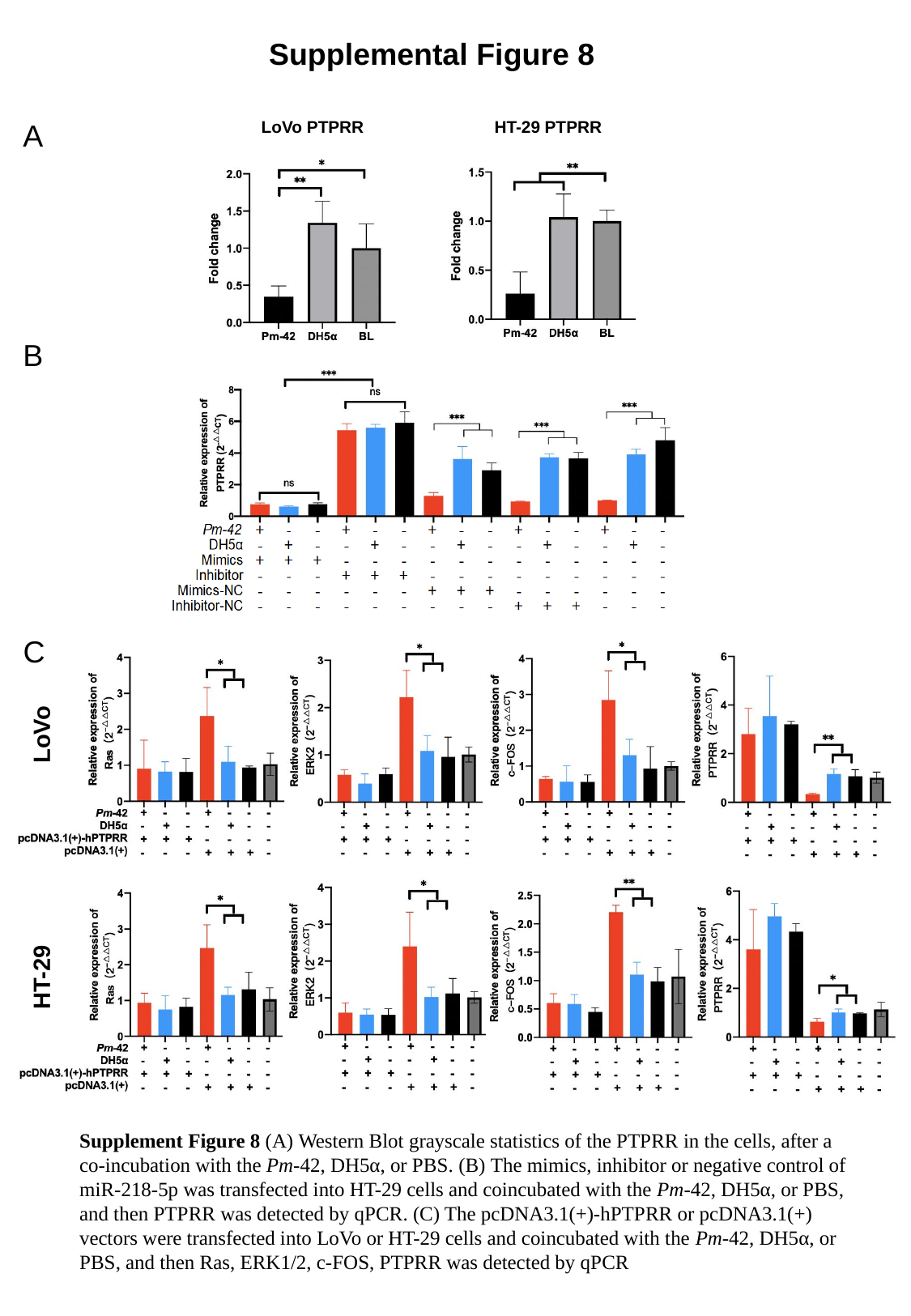

Supplemental Figure 8
A
LoVo PTPRR
HT-29 PTPRR
B
C
LoVo
HT-29
Supplement Figure 8 (A) Western Blot grayscale statistics of the PTPRR in the cells, after a co-incubation with the Pm-42, DH5α, or PBS. (B) The mimics, inhibitor or negative control of miR-218-5p was transfected into HT-29 cells and coincubated with the Pm-42, DH5α, or PBS, and then PTPRR was detected by qPCR. (C) The pcDNA3.1(+)-hPTPRR or pcDNA3.1(+) vectors were transfected into LoVo or HT-29 cells and coincubated with the Pm-42, DH5α, or PBS, and then Ras, ERK1/2, c-FOS, PTPRR was detected by qPCR

## Slide 9
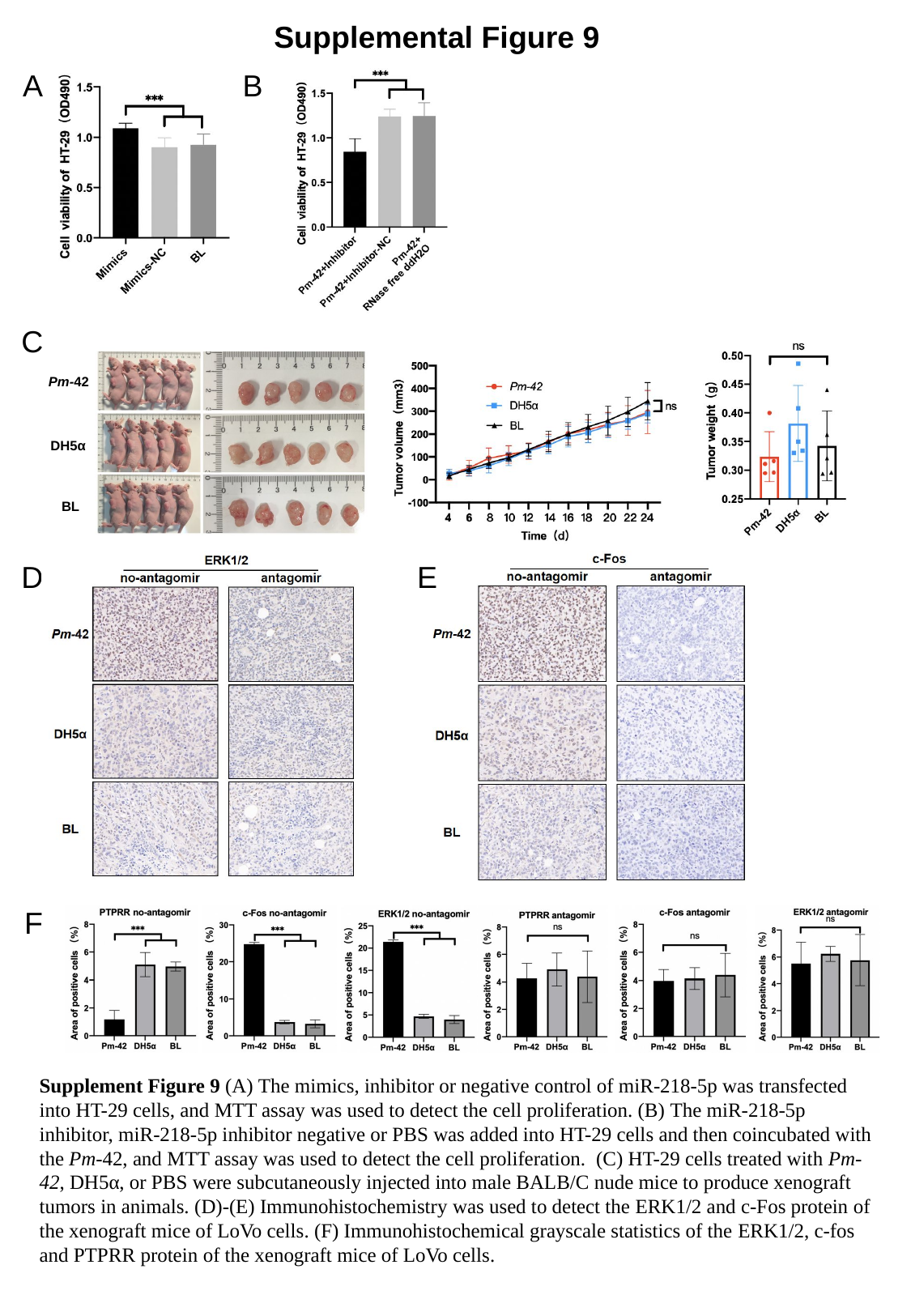

Supplemental Figure 9
A
B
C
Pm-42
DH5α
BL
D
E
F
Supplement Figure 9 (A) The mimics, inhibitor or negative control of miR-218-5p was transfected into HT-29 cells, and MTT assay was used to detect the cell proliferation. (B) The miR-218-5p inhibitor, miR-218-5p inhibitor negative or PBS was added into HT-29 cells and then coincubated with the Pm-42, and MTT assay was used to detect the cell proliferation. (C) HT-29 cells treated with Pm-42, DH5α, or PBS were subcutaneously injected into male BALB/C nude mice to produce xenograft tumors in animals. (D)-(E) Immunohistochemistry was used to detect the ERK1/2 and c-Fos protein of the xenograft mice of LoVo cells. (F) Immunohistochemical grayscale statistics of the ERK1/2, c-fos and PTPRR protein of the xenograft mice of LoVo cells.

## Slide 10
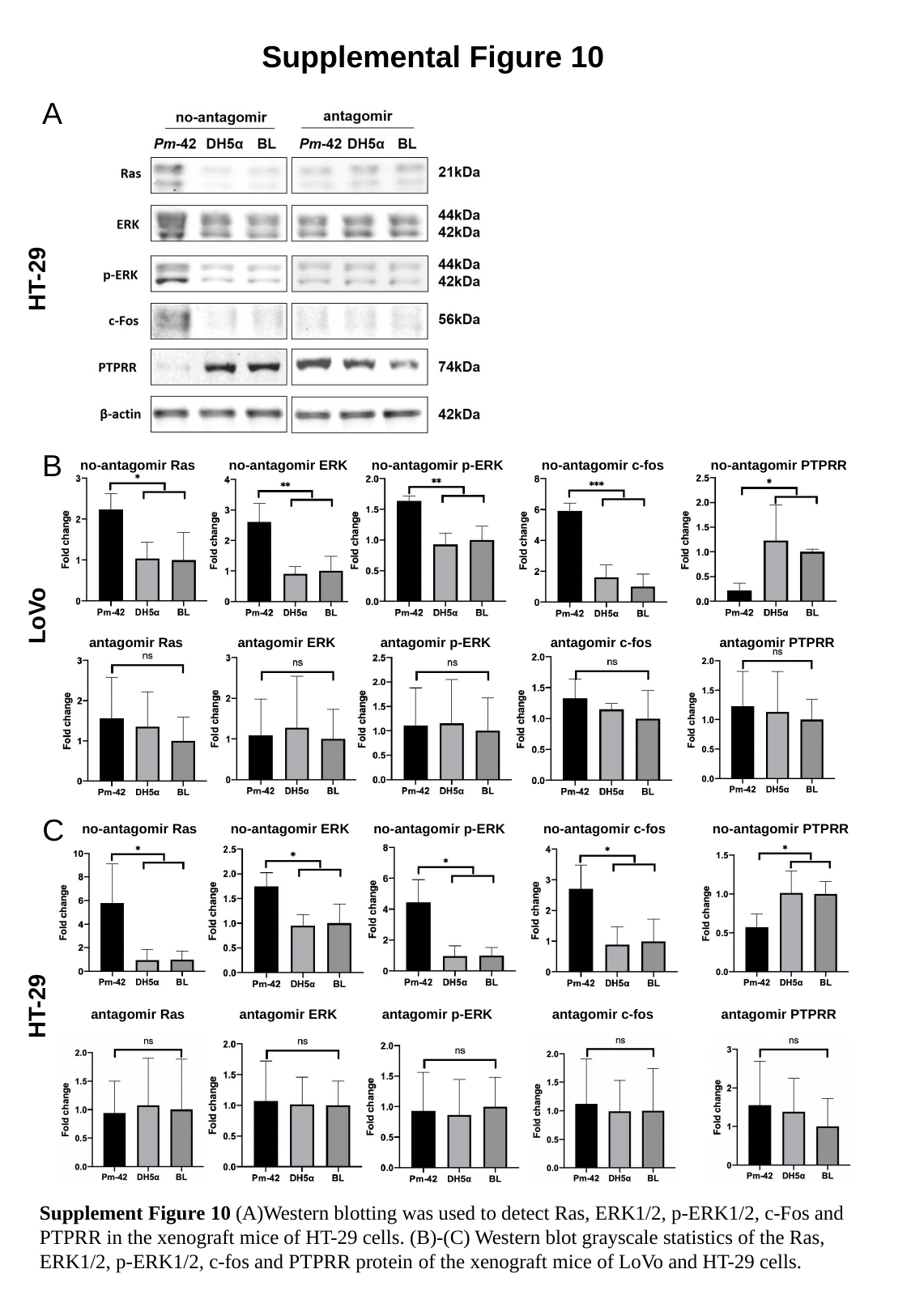

Supplemental Figure 10
A
HT-29
B
no-antagomir Ras
no-antagomir ERK
no-antagomir p-ERK
no-antagomir c-fos
no-antagomir PTPRR
LoVo
antagomir Ras
antagomir ERK
antagomir p-ERK
antagomir c-fos
antagomir PTPRR
C
no-antagomir Ras
no-antagomir ERK
no-antagomir p-ERK
no-antagomir c-fos
no-antagomir PTPRR
HT-29
antagomir Ras
antagomir ERK
antagomir p-ERK
antagomir c-fos
antagomir PTPRR
Supplement Figure 10 (A)Western blotting was used to detect Ras, ERK1/2, p-ERK1/2, c-Fos and PTPRR in the xenograft mice of HT-29 cells. (B)-(C) Western blot grayscale statistics of the Ras, ERK1/2, p-ERK1/2, c-fos and PTPRR protein of the xenograft mice of LoVo and HT-29 cells.

## Slide 11
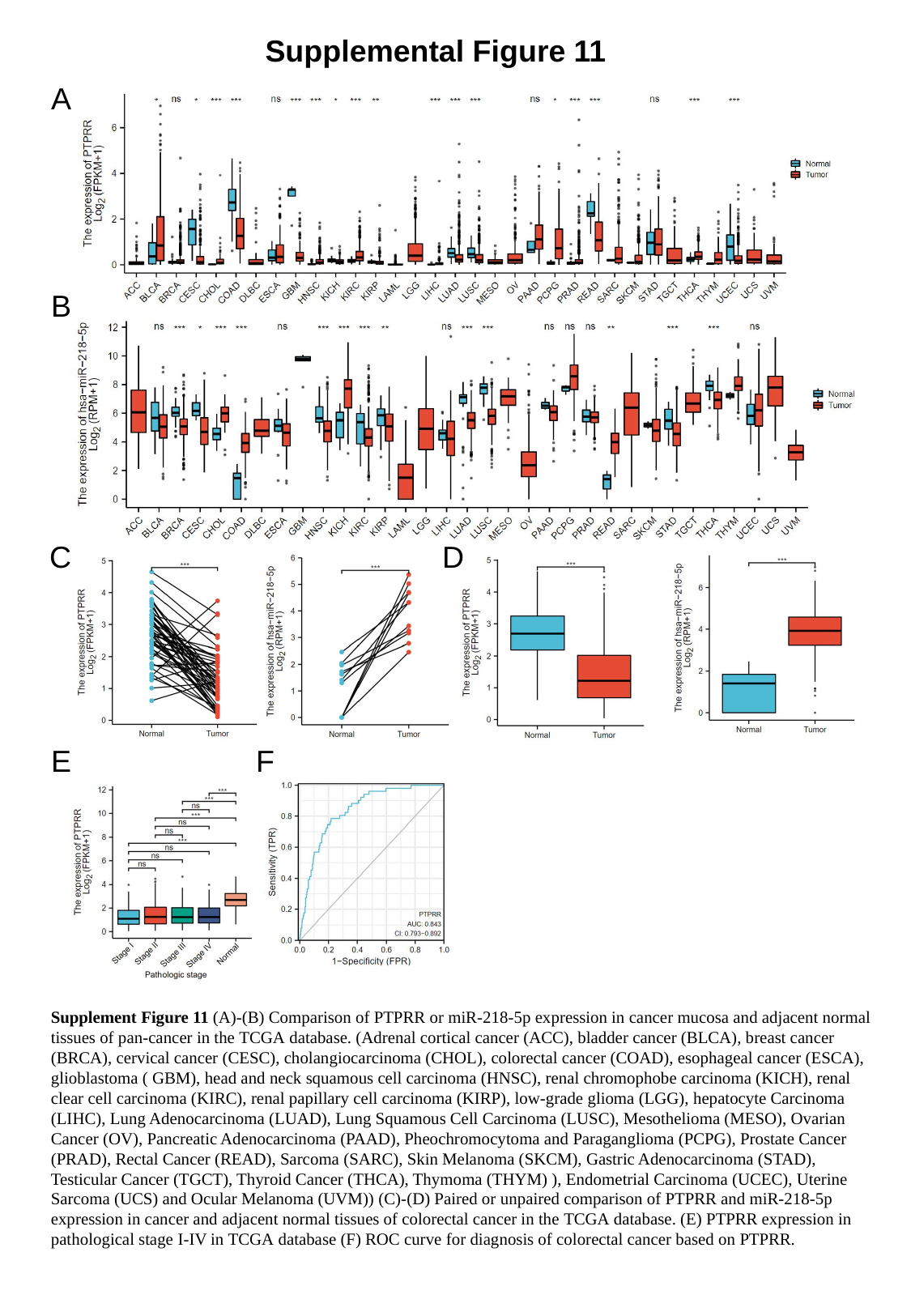

Supplemental Figure 11
A
B
C
D
E
F
Supplement Figure 11 (A)-(B) Comparison of PTPRR or miR-218-5p expression in cancer mucosa and adjacent normal tissues of pan-cancer in the TCGA database. (Adrenal cortical cancer (ACC), bladder cancer (BLCA), breast cancer (BRCA), cervical cancer (CESC), cholangiocarcinoma (CHOL), colorectal cancer (COAD), esophageal cancer (ESCA), glioblastoma ( GBM), head and neck squamous cell carcinoma (HNSC), renal chromophobe carcinoma (KICH), renal clear cell carcinoma (KIRC), renal papillary cell carcinoma (KIRP), low-grade glioma (LGG), hepatocyte Carcinoma (LIHC), Lung Adenocarcinoma (LUAD), Lung Squamous Cell Carcinoma (LUSC), Mesothelioma (MESO), Ovarian Cancer (OV), Pancreatic Adenocarcinoma (PAAD), Pheochromocytoma and Paraganglioma (PCPG), Prostate Cancer (PRAD), Rectal Cancer (READ), Sarcoma (SARC), Skin Melanoma (SKCM), Gastric Adenocarcinoma (STAD), Testicular Cancer (TGCT), Thyroid Cancer (THCA), Thymoma (THYM) ), Endometrial Carcinoma (UCEC), Uterine Sarcoma (UCS) and Ocular Melanoma (UVM)) (C)-(D) Paired or unpaired comparison of PTPRR and miR-218-5p expression in cancer and adjacent normal tissues of colorectal cancer in the TCGA database. (E) PTPRR expression in pathological stage I-IV in TCGA database (F) ROC curve for diagnosis of colorectal cancer based on PTPRR.
